# Microbiome-aware oral drug delivery: excipient–microbiome interactions and nanocarrier design rules for predictable formulation performance

**DOI:** 10.3389/fphar.2026.1942291

**Published:** 2026-09-25

**Authors:** Mohamed Sedeek, Ahmed Ayoub, Essam Hany, Ahmed Bahaa, Amr Bahr, Osama Saher, Sadek Ahmed

**Affiliations:** 1 Faculty of Pharmacy, Egypt Japan University of Science and Technology (E-JUST), New Borg El-Arab City, Egypt; 2 Department of Pharmaceutics and Industrial Pharmacy, Faculty of Pharmacy, Cairo University, Cairo, Egypt; 3 Department of Laboratory Medicine, Karolinska Institute, Stockholm, Sweden; 4 Department of Cellular Therapy and Allogeneic Stem Cell Transplantation (CAST), Karolinska University Hospital Huddinge and Karolinska Comprehensive Cancer Center, Stockholm, Sweden

**Keywords:** colon targeting, formulation design, gut microbiome, microbiota-responsive delivery, nanocarriers, oral drug delivery, pharmaceutical excipients

## Abstract

Oral drug delivery remains the predominant administration route, but formulation strategies rarely account systematically for the gut microbiome. The microbiome can alter drug metabolism, excipient behavior, nanocarrier interactions, and interpatient variability. This review integrates evidence on microbial drug metabolism, excipient–microbiome interactions, nanocarrier engineering, microbiota-responsive delivery, and experimental models. Clinically relevant examples include digoxin inactivation by *Eggerthella lenta*, bacterial levodopa metabolism, and microbial beta-glucuronidase-mediated irinotecan toxicity. Microbiota-degradable polysaccharides may serve as colon-targeted release triggers, whereas some surfactants and polymers may create context-dependent microbiome or barrier risks. Nanocarrier size, charge, coating, mucoadhesion, mucus penetration, biodegradability, enzyme responsiveness, and surfactant burden are treated as microbiome-interface variables. We propose an authors’ evidence-informed framework comprising an excipient classification, nanocarrier design rules, microbiota-responsive decision logic, and a tiered testing roadmap. These proposals are not established regulatory classifications; they are intended to support more predictable and patient-relevant oral formulation development.

## Graphical Abstract

**Figure:**
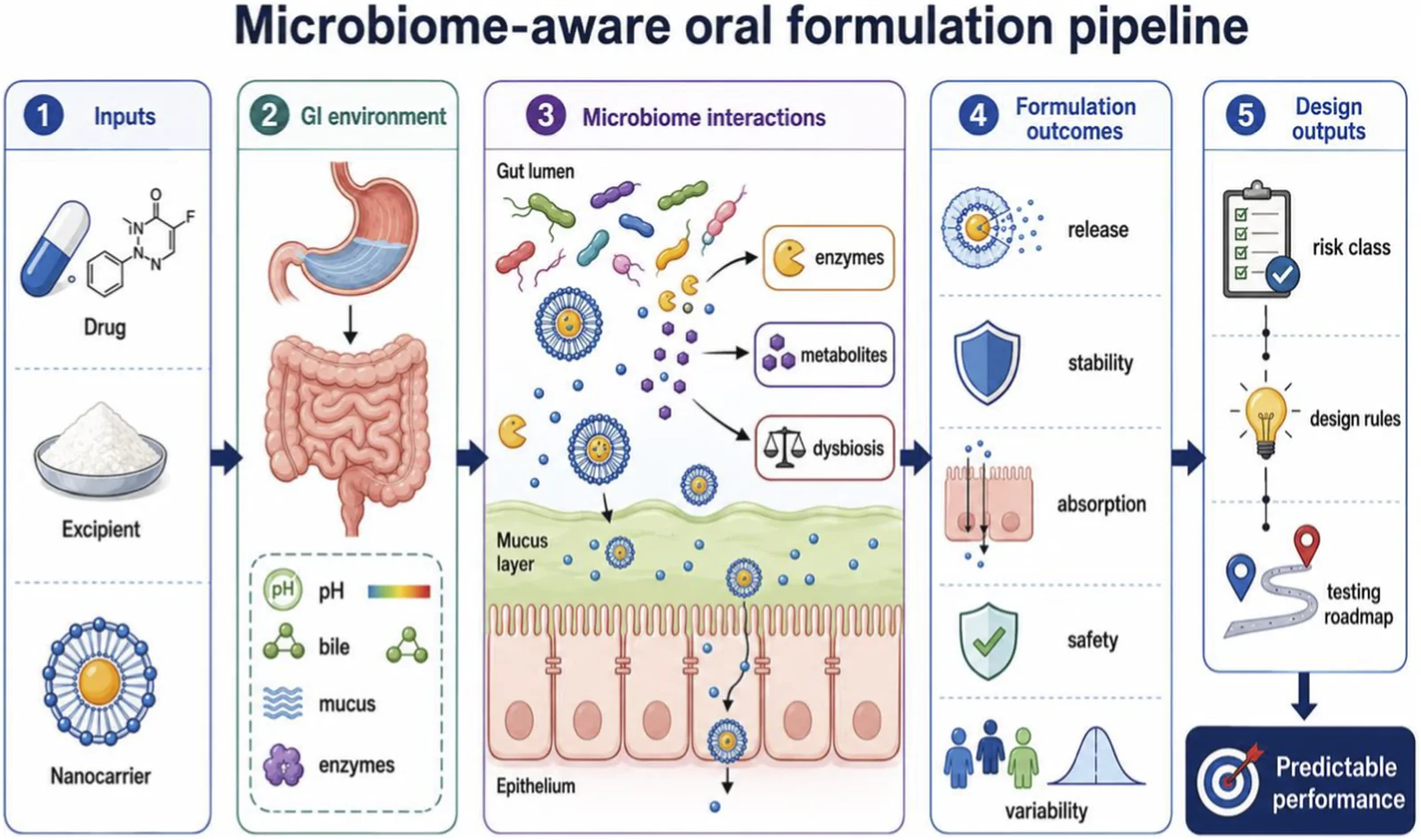

## Highlights

- Reframes the gut microbiome as a formulation-relevant variable in oral drug delivery.
- Proposes an authors’ perspective classification of excipients by microbiome-relevant risk and opportunity.
- Proposes nanocarrier design rules based on microbiome-interface properties.
- Proposes decision logic for microbiota-responsive oral dosage forms.
- Outlines tiered testing endpoints for microbiome-aware formulation development.

## 1 Introduction

Oral drug delivery remains the most widely preferred route for pharmaceutical development because of its convenience, patient acceptability, scalability, and suitability for long-term therapy. However, achieving predictable oral formulation performance remains difficult because the gastrointestinal (GI) tract is complex. Oral dosage forms must navigate dynamic pH gradients, gastric emptying, intestinal transit, regional fluid composition, mucus, digestive enzymes, epithelial permeability barriers, and first-pass metabolism (Hua, 2020).

Food state and gastrointestinal pH can further alter drug dissolution, stability, and absorption, especially for drugs with pH-dependent solubility or permeability (Abuhelwa et al., 2017). These classical variables remain central to oral formulation design, but they do not fully capture the chemical and biological complexity of the intestinal environment (Ting et al., 2022).

One increasingly important layer of this complexity is the gut microbiome. Gut bacteria can chemically modify many orally administered drugs (Zimmermann et al., 2019). Individual gut microbial communities can also differ in their drug-metabolizing capacity, suggesting that microbiome composition may contribute to patient-to-patient variability in oral drug exposure (Javdan et al., 2020). Mechanistic examples make this concept clinically and formulation-relevant. *Eggerthella lenta* can inactivate digoxin, showing that microbial metabolism may reduce active drug exposure (Haiser et al., 2013). Gut bacterial enzymes can metabolize levodopa before it reaches systemic circulation, potentially reducing availability and contributing to variable response (Rekdal et al., 2019). Bacterial beta-glucuronidase activity can also reactivate drug metabolites and contribute to irinotecan-associated intestinal toxicity (Tsunoda et al., 2021). These examples show that microbiomes may influence oral drug stability, activity, exposure, and safety.

Microbial drug metabolism creates both risks and opportunities for formulation design. If a drug is susceptible to microbial degradation before absorption, the formulation may need to protect it during gastrointestinal transit (Hua, 2020). If microbial metabolism generates toxic or irritating products, the formulation may need to reduce local intestinal exposure. Conversely, if microbial enzymes are sufficiently localized and predictable, they may be exploited as triggers for colon-targeted or microbiota-responsive drug release (Qiu et al., 2023). Thus, the microbiome should not be treated only as a host biology topic; it can function as a formulation-relevant barrier, metabolic compartment, and release trigger (Gebrayel et al., 2022).

This microbiome-aware view also challenges the conventional separation between active pharmaceutical ingredients and inactive formulation components. Pharmaceutical excipients may directly interact with gut microbes. Some food and drug additives can inhibit intestinal drug transport, indicating that formulation components may influence absorption-related processes (Zou et al., 2020). PEG400 has been associated with changes in gut microbiota and fecal metabolomic profiles in mice, suggesting that at least some excipients may have microbiome-relevant effects under specific exposure conditions (Zhao M. et al., 2023). These findings do not imply that all excipients are harmful or disrupt the microbiome. Rather, they support a risk-based approach in which excipient effects are interpreted according to compound identity, dose, exposure duration, formulation context, and patient vulnerability.

Oral nanocarriers add another layer to this formulation–microbiome interface. Nanomaterial effects on the gut microbiota have been reported to depend on size, dose, exposure duration, and functional groups (Utembe et al., 2022). Oral nanomedicine systems may also interact with intestinal barrier functions and the gut microbiome, especially when designed for local gastrointestinal action (Lee et al., 2021). Mucus is a critical interface for oral nanocarriers because particles must adhere to it, penetrate it, or avoid excessive trapping depending on the intended delivery goal (Wang et al., 2023). Therefore, nanocarrier size, surface charge, hydrophobicity, surface coating, mucoadhesion, mucus penetration, biodegradability, enzyme responsiveness, and surfactant load should be considered both as pharmaceutical design variables and as microbiome-interface variables (Garbati et al., 2024).

Several related bodies of literature have addressed parts of this problem. Reviews on pharmaceutical excipients have highlighted possible excipient–microbiota interactions (Subramaniam et al., 2023a). Reviews on nanomaterials have summarized how physicochemical properties may shape gut microbiota effects (Utembe et al., 2022). Some reviews on oral drug bioavailability describe microbiota as a factor that may influence oral drug fate (Zhang et al., 2021), and other reviews on microbiota-sensitive polysaccharide systems have discussed microbial degradation as a colon-targeting mechanism (Azehaf et al., 2023). However, these areas often remain separated. Drug-microbiome studies frequently emphasize metabolism and response variability (The Integrative HMP iHMP Research Network Consortium et al., 2019). Excipient studies often focus on microbiota disruption or additive safety (Subramaniam et al., 2023a). Nanocarrier studies often prioritize delivery performance (Hocaoğlu et al., 2026). Colon-targeted delivery studies often describe microbial triggers without fully integrating patient microbiome variability, excipient risk, nanocarrier interface properties, and microbiome-aware testing (Chen et al., 2025).

Rather than providing another general overview of gut microbiome biology, the review integrates evidence on microbial drug metabolism, excipient–microbiome interactions, nanocarrier–microbiome interfaces, microbiota-responsive release mechanisms, and experimental testing models. Its practical objective is to translate this evidence into four formulation outputs: an excipient–microbiome-risk classification, nanocarrier design rules at the gut microbiome interface, a microbiota-responsive oral delivery decision tree, and a minimum testing roadmap for microbiome-aware oral formulations. These four outputs are presented as the authors’ proposed, evidence-informed tools rather than established regulatory classifications or mandatory development standards.

Unlike prior reviews that treat microbial drug metabolism, excipient effects, nanomaterial safety, or colon targeting as largely separate topics, this review integrates these domains into a formulation-centered framework linking evidence strength to excipient selection, nanocarrier design, trigger choice, and testing strategy.

By adopting this formulation-centered perspective, microbiome-aware oral drug delivery can move beyond descriptive associations toward more predictable design logic (Alexander et al., 2026). The key question is not simply whether the gut microbiome affects oral drugs but when microbiome effects are sufficiently relevant to influence excipient selection, nanocarrier engineering, release-trigger choice, model selection, safety testing, and patient-specific translation. This positions the gut microbiome as a critical interface connecting drug stability, release, absorption, safety, and interindividual variability in oral dosage-form performance. Figure 1 illustrates the bidirectional formulation–microbiome interaction map.

**FIGURE 1:**
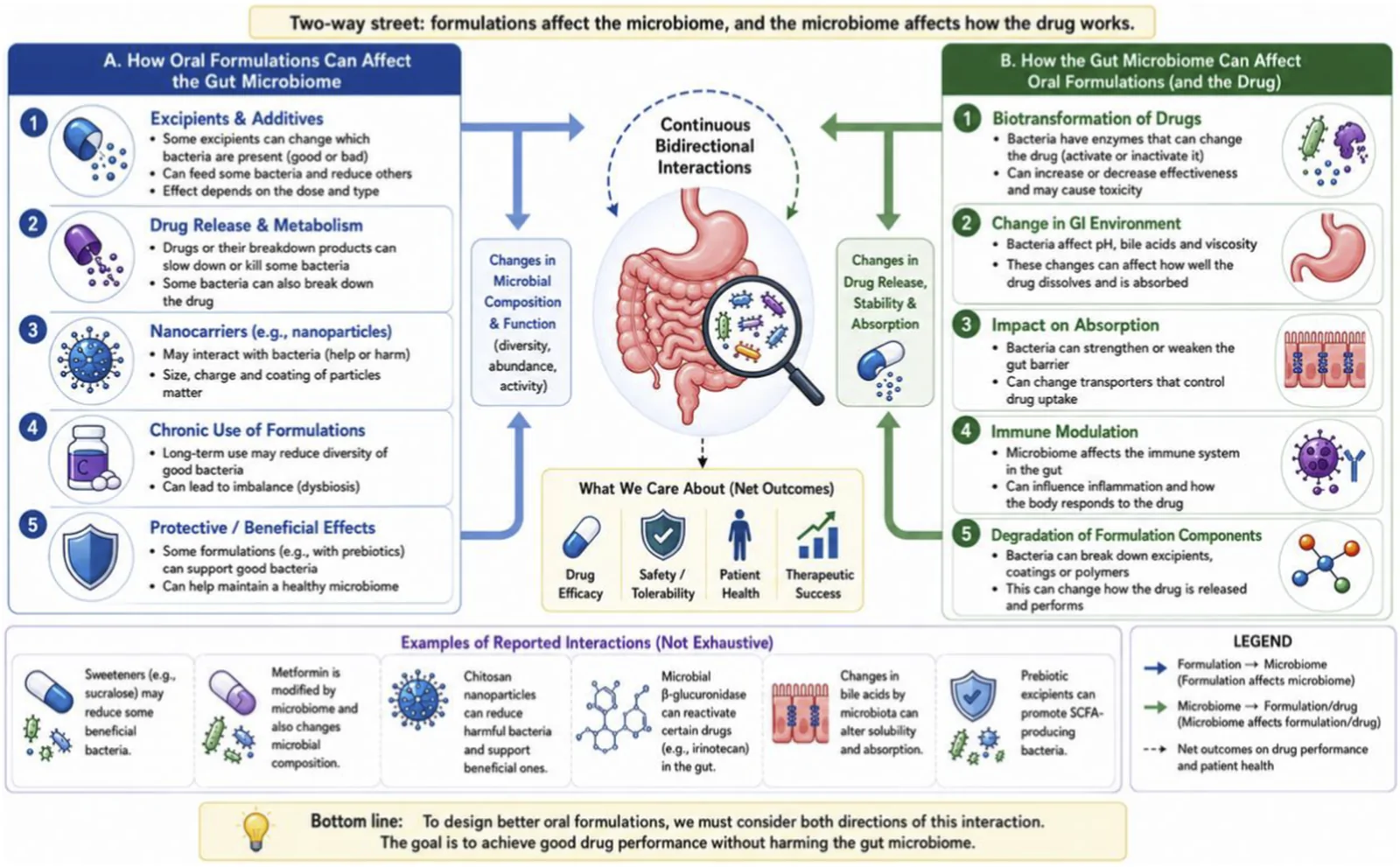
Bidirectional formulation–microbiome interaction map. Oral formulations may alter microbial composition and function through excipients, additives, drug release patterns, nanocarriers, and chronic exposure, while the gut microbiome can affect drug biotransformation, the gastrointestinal environment, absorption, immune responses, and degradation of formulation components. The net outcome influences efficacy, safety, patient health, and therapeutic success.

## 2 Gut microbiome as a formulation-relevant barrier and modulator

The gut microbiome can be considered a formulation-relevant barrier and modulator because it introduces metabolic, enzymatic, physicochemical, and biological variability into the gastrointestinal environment (Jyoti and Dey, 2025). Unlike classical barriers such as pH, mucus, epithelial permeability, and enzymatic activity, which vary within relatively predictable physiological ranges, the microbiome is highly dynamic in composition and function and is profoundly patient-specific (Aggarwal et al., 2023).

### 2.1 Microbial metabolism of orally administered drugs

Microbial drug metabolism is one of the clearest mechanisms linking the gut microbiome to oral drug performance. A broad experimental screen demonstrated that gut bacteria can chemically modify numerous orally administered drugs, establishing the microbiome as an active drug-metabolizing environment within the gastrointestinal tract (Zimmermann et al., 2019). Such transformations may occur before absorption, during prolonged intestinal transit, or following biliary excretion of drug metabolites. Importantly, personalized *ex vivo* studies have shown that drug-metabolizing capacity varies substantially among individual microbial communities, indicating that microbial metabolism is both a drug-specific and patient-specific determinant of oral drug exposure (Javdan et al., 2020). Several well-characterized examples illustrate the potential consequences for oral delivery. *Eggerthella lenta* can reduce and inactivate digoxin, thereby decreasing active drug exposure (Haiser et al., 2013). Gut bacterial tyrosine decarboxylases metabolize levodopa, reducing the amount of drug available for systemic absorption and contributing to variability in Parkinson disease treatment outcomes (Van Kessel et al., 2019; Rekdal et al., 2019). In contrast, bacterial β-glucuronidases can reactivate irinotecan metabolites in the intestine, promoting local gastrointestinal toxicity (Wallace et al., 2010). Together, these examples demonstrate that microbial activity can influence oral drug fate through drug inactivation, presystemic metabolism, metabolite reactivation, and toxicity modulation (Peng et al., 2007).

Microbial metabolism can also be intentionally exploited for local drug activation. Azo-bonded prodrugs of 5-aminosalicylic acid, including sulfasalazine, balsalazide, and olsalazine, depend on bacterial azo reduction to cleave the azo bond and release active 5-aminosalicylic acid in the distal intestine. Comparative fermentation studies using human fecal microbiota show that these prodrugs differ in degradation rates, indicating that microbial enzyme availability alone does not determine release kinetics; the broader molecular structure of the prodrug also influences microbiota-mediated activation (Sousa et al., 2014). This example demonstrates that microbial metabolism may serve as a deliberately engineered release mechanism rather than only a source of pharmacokinetic variability.

Conversely, microbial transformation may reduce systemic drug activity. Commensal gut bacteria, including *Faecalibacterium prausnitzii* and other members of the order Clostridiales, have been shown to convert tacrolimus through C-9 keto reduction to a metabolite designated M1. This metabolite was approximately 15-fold less potent than tacrolimus in an immunosuppressive activity assay, and its formation was demonstrated in human fecal incubations and stool samples from transplant recipients receiving oral tacrolimus (Guo et al., 2019). Because tacrolimus has a narrow therapeutic index and highly variable oral exposure, microbial conversion represents a plausible additional contributor to patient-to-patient differences in dose requirements, although its quantitative clinical contribution requires further validation.

Microbial drug metabolism may also generate metabolites that interact with host metabolic pathways and produce severe toxicity. Sorivudine provides a clinically important example: intestinal microbiota convert sorivudine to bromovinyluracil, which is subsequently absorbed and causes mechanism-based inactivation of hepatic dihydropyrimidine dehydrogenase. This prevents normal degradation of 5-fluorouracil. This reaction contributed to severe and fatal toxicity in patients who received sorivudine together with oral fluoropyrimidine prodrugs (Okuda et al., 1997). This case demonstrates that microbial metabolism of one orally administered drug may indirectly alter the systemic disposition and toxicity of a second drug.

The formulation relevance of microbial metabolism depends on the balance between microbial transformation and drug absorption, along with the biological activity of the resulting metabolites. Consequently, microbial metabolism should be considered a risk assessment factor rather than a universal limitation of oral delivery (Pedroza Matute and Iyavoo, 2023). This consideration is particularly important for drugs with narrow therapeutic windows, known microbial susceptibility, or highly variable clinical responses. The literature on drug–microbiota interactions also suggests that differences in microbiome composition and enzymatic capacity may contribute to variability in drug disposition, efficacy, and adverse effects among patients (Zhao Q. et al., 2023).

From a formulation perspective, microbiome-sensitive drugs may require strategies that reduce microbial exposure prior to absorption, alter the site of drug release, shorten intestinal residence time, or otherwise account for microbiome-dependent metabolism (Zhao Q. et al., 2023). Conversely, delivery systems that intentionally exploit microbial enzymes as release triggers must consider interindividual variability in microbial activity, which may affect release kinetics and therapeutic consistency (Kamath et al., 2025). These challenges highlight a potential future role for microbiome-informed formulation design, particularly for microbiota-responsive colon-targeted systems and drugs exhibiting substantial response variability (Javdan et al., 2020).

### 2.2 Microbial enzymes as determinants of drug fate and formulation triggers

Beyond their role in pharmacokinetic variability, microbial enzymes can be actively exploited as formulation triggers. Colon-targeted systems have long exploited the fact that the distal gut contains dense microbial communities that can degrade specific chemical bonds or polymeric structures (Miller et al., 2025). Natural polysaccharides are especially important because they can act as substrates for enzymes produced by colonic microbiota (Azehaf et al., 2023). This supports using pectin, chitosan, alginate, dextran, guar gum, inulin, and related materials in microbiota-sensitive delivery systems (Azehaf et al., 2023).

Microbial enzymes should therefore be considered in two related but distinct roles: as determinants of drug fate and as formulation triggers. Their effects are not intrinsically beneficial or detrimental; they depend on the substrate, the biological activity of the product, the site and timing of transformation, and whether the pathway is intentionally incorporated into the therapeutic design.

A beneficial transformation occurs when bacterial metabolism is required to activate a drug. Colonic bacterial azoreductases cleave the azo bonds of sulfasalazine, balsalazide, and olsalazine, releasing active 5-aminosalicylic acid in the distal intestine. Human fecal fermentation studies show that the three prodrugs are degraded at different rates, indicating that the surrounding molecular structure, along with the azo bond itself, influences microbial accessibility and activation kinetics (Sousa et al., 2014). This pathway has relatively strong translational support because microbiota-dependent activation is an established component of the therapeutic design, although individual release rates may still vary.

Detrimental transformation can involve reactivation of a toxic metabolite. Gut bacterial beta-glucuronidases hydrolyze the inactive irinotecan conjugate SN-38 glucuronide and regenerate active SN-38 in the intestinal lumen, increasing local epithelial injury and diarrhea. Selective inhibition of bacterial beta-glucuronidases reduced irinotecan-associated toxicity in mice without eliminating the antitumor effect (Wallace et al., 2010). This provides strong mechanistic and preclinical evidence for the enzyme-toxicity relationship, while the quantitative contribution of this pathway to interpatient toxicity remains less directly established.

Presystemic microbial metabolism can also reduce active drug availability. Bacterial tyrosine decarboxylases convert levodopa to dopamine in the intestinal lumen; experimental work combined bacterial conversion assays, patient-associated gene-abundance data, and rat pharmacokinetic evidence linking higher enzyme abundance to lower levodopa availability (Van Kessel et al., 2019). The cardiac glycoside reductase system of *E. lenta* reduces digoxin to the less active metabolite dihydrodigoxin, supported by culture-based, genomic, and gnotobiotic-mouse studies (Haiser et al., 2013). These pathways are mechanistically well supported, but prospective clinical evidence for microbiome-guided dose or formulation selection remains limited.

For formulation development, evidence should therefore be interpreted at three levels: confirmation that the relevant microbial activity transforms the intended substrate; demonstration that the transformation produces the expected release or pharmacological consequence; and evidence that the process remains sufficiently consistent across microbiomes and clinically relevant disease states. Established prodrug activation should be distinguished from formulation concepts supported mainly by *in vitro* enzyme systems, fecal incubations, or preclinical models. Where enzyme activity is variable, multi-trigger designs may reduce risk, but they should be described as proposed risk-mitigation strategies until validated in translational models.

Azo chemistry provides another classic example of microbial enzyme-responsive delivery. Colon-targeted delivery reviews describe azo-based systems as strategies that exploit the colonic microflora and bacterial enzymes for site-specific drug release (Amidon et al., 2015; Li et al., 2026). Enzyme-responsive nanocarriers have also been designed using microbial-enzyme-sensitive layers, such as cellulase-responsive solid lipid nanoparticles for colonic budesonide release (Zhang et al., 2023). These examples show how microbial activity can be converted into a formulation design tool.

However, microbiota-responsive release is only useful if the trigger is sufficiently reliable (Ma et al., 2024). Microbial enzyme abundance may vary by patient, disease state, diet, antibiotic exposure history, and intestinal region. In active Crohn’s disease, bacterial enzymes used for colon-specific delivery have been reported to decrease, raising concerns about the reliability of enzyme-triggered release in inflamed or dysbiotic states (Sarmah et al., 2026). Consequently, microbiota-responsive formulations should be developed with recognition that the gut microbiome is highly heterogeneous between individuals (Azehaf et al., 2023). For applications where microbial composition and activity vary substantially, multi-trigger systems incorporating pH-, transit time-, and microbiota-responsive elements may be more robust than reliance on microbial triggers alone (Carrette et al., 1995).

### 2.3 Microbiome effects on mucus, epithelial barrier, and absorption

The microbiome can also influence oral drug delivery indirectly through the mucus layer, epithelial barrier, microbial metabolites, and intestinal transport processes. Gastrointestinal mucus is a critical interface between luminal contents, bacteria, drug molecules, drug carriers, and epithelial tissues (Wang et al., 2023). Any oral nanocarrier or particulate formulation must therefore interact with mucus before reaching the epithelium, whether the goal is local retention, mucus penetration, or epithelial absorption (Subramanian et al., 2022). Microbiome-driven changes in mucus structure or composition could therefore alter formulation residence time, diffusion, and access to the epithelial surface.

Microbial metabolites may also affect epithelial barrier function. Butyrate, for example, can influence intestinal barrier properties in a concentration-dependent manner in epithelial models (Adams et al., 2024). This shows that microbial metabolites are not merely biomarkers of microbiome activity; they may also modify the absorptive environment encountered by oral formulations. Reviews of drug–microbiota interactions also suggest that microbiomes can affect intestinal drug transporters and barrier-related processes (Zhao Q. et al., 2023). These mechanisms are formulation-relevant because permeability enhancers, surfactants, cationic nanoparticles, and mucoadhesive systems all depend on the condition of the mucus–epithelium interface.

Overall, the gut microbiome can modulate oral drug delivery through direct drug metabolism, patient-specific metabolic capacity, microbial enzyme-triggered release, mucus interactions, barrier modulation, and metabolite-mediated effects. The strongest evidence exists for microbial drug metabolism and selected enzyme-responsive delivery strategies, while microbiome effects on absorption, mucus, transporters, and barrier function remain more context dependent and require stronger formulation-specific validation (Al-Btoosh et al., 2026). This evidence supports treating the microbiome as a formulation-relevant variable but not as a universal determinant of oral performance. The next design question is how formulation components themselves, especially excipients, interact with this microbial environment. Table 1 summarizes the main barriers and mechanisms relating to the microbiome.

**TABLE 1:** Gastrointestinal barriers and microbiome-related mechanisms relevant to oral formulation.

Barrier	Mechanism/description	Formulation relevance	Reference
Dynamic pH gradients	• Stomach: approximately pH 1–2 • Small intestine: approximately pH 6–7; colon: approximately pH 7–8	• Controls ionization, dissolution, stability, and absorption • Guides release-site selection	Abuhelwa et al. (2017), Hua (2020)
Gastric emptying and intestinal transit	• Gastric emptying and intestinal transit are variable • Food and disease can modify transit	• Determines time available for dissolution and absorption • Affects timing of site-specific release	Abuhelwa et al. (2017), Hua (2020)
Mucus layer	• Viscoelastic coating over the intestinal epithelium • Size- and charge-selective barrier influenced by microbiota	• Requires a defined adhesion or penetration strategy • Charge and hydrophobicity affect mucus trapping	Subramanian et al. (2022), Wang et al. (2023)
Epithelial permeability	• Tight junctions restrict paracellular transport • Transcellular uptake depends on drug and transporter properties	• Targeted by permeation enhancers, surfactants, and cationic carriers • Microbial metabolites can alter barrier integrity	Zhao Q. et al. (2023)
Digestive enzymes	• Host proteases, lipases, and related enzymes degrade susceptible materials • Exposure occurs throughout gastrointestinal transit	• Labile drugs and excipients may require protection • Carrier biodegradability and enzyme responsiveness require control	Hua (2020)
First-pass metabolism	• Portal absorption exposes drugs to hepatic metabolism • Cytochrome P450 and conjugation can reduce bioavailability	• Influences dose and prodrug selection • Supports lymphatic-targeting strategies for selected drugs	Hua (2020)
Microbial drug metabolism	• Bacteria transform drugs by reduction, hydrolysis, decarboxylation, and related reactions • Capacity is community- and patient-dependent	• May inactivate drugs, reduce availability, or reactivate toxic metabolites • Important for narrow-therapeutic-index drugs	Javdan et al. (2020), Zimmermann et al. (2019)
Patient-specific microbial variability	• Microbial composition and enzyme activity vary between patients • Variation reflects host, disease, diet, and exposure factors	• Contributes to variable exposure, efficacy, and adverse effects • Challenges uniform release and dose assumptions	Javdan et al. (2020), Zhao Q. et al. (2023)
Microbial modulation of drug transporters and barrier	• Microbial metabolites influence tight junctions and transporters • Effects may change permeability and absorption conditions	• Alters the absorptive environment independently of the dosage form • Relevant to permeability-dependent formulations	Zhao Q. et al. (2023)
Excipient–microbiome interactions	• Excipients may affect microbes, transporters, metabolites, or barrier function • Effects depend on compound and exposure	• Use dose-, duration-, and patient-specific risk assessment • Do not assume inactive ingredients are microbiome-neutral	Zhao M. et al. (2023), Zou et al. (2020)
Unreliable enzyme triggers in dysbiosis	• Disease or antibiotics may reduce microbial trigger activity • Active Crohn’s disease is a relevant example	• Microbiota-only release may become delayed or incomplete • Backup pH or time triggers may improve robustness	Azehaf et al. (2023)

## 3 Excipient–microbiome interactions

Pharmaceutical excipients are traditionally selected to improve manufacturability, stability, palatability, solubility, permeability, coating performance, or release control. In regulatory and formulation language, they are often described as inactive ingredients. However, “inactive” should not be interpreted as universally biologically inert. For microbiome-aware oral drug delivery, excipients deserve attention because they often make up higher mass fractions than the active drug, may be repeatedly administered in chronic therapies, and directly encounter the intestinal lumen. A review focused on pharmaceutical excipients reported that orally administered excipients may directly interact with gut microbes (Subramaniam et al., 2023a). This does not mean that all excipients disrupt the microbiome. Rather, excipient selection should be interpreted through a risk-based lens that considers compound identity, dose, duration, formulation type, and patient context.

### 3.1 Excipients are not always biologically inert

The strongest formulation-relevant argument is that excipients can influence oral drug performance through mechanisms beyond their traditional technological functions. A systematic screening study demonstrated that several commonly used food and pharmaceutical additives inhibit intestinal drug transporters, indicating that formulation components can directly affect absorption-related pathways independent of the active pharmaceutical ingredient (Metry and Polli, 2022; Zou et al., 2020). These effects do not necessarily require microbiome involvement to be clinically relevant; excipients may alter drug exposure by modulating transporters, membrane properties, mucus interactions, solubilization, or epithelial barrier function (Burke and Li, 2025).

Several examples illustrate these mechanisms. Certain surfactants and solubilizing agents, including polysorbates and polyethylene glycol derivatives, have been reported to modify membrane permeability and transporter activity, thereby influencing intestinal drug absorption. Medium-chain fatty acids such as sodium caprate can transiently open tight junctions and enhance paracellular transport, a property exploited in oral peptide delivery systems (Twarog et al., 2019).

Bile salt-based excipients and lipid formulations can increase drug solubilization and promote lymphatic transport, thereby altering oral bioavailability and first-pass metabolism (Fahmy et al., 2026; Hua, 2020). In addition, mucoadhesive polymers such as chitosan may increase epithelial residence time while simultaneously affecting mucus structure and barrier properties (Basrowi et al., 2020).

The microbiome adds an additional layer of complexity because many excipients are themselves substrates for microbial metabolism (Bakkeren et al., 2025). For example, polysaccharide-based excipients, including pectin, guar gum, dextran, and resistant starch, can be fermented by colonic bacteria, altering local pH, short-chain fatty acid production, and polymer degradation kinetics that influence drug release behavior (Zeng et al., 2025). Likewise, microbiota-responsive colon-targeted systems intentionally exploit bacterial enzymes to degrade polysaccharide carriers, making formulation performance dependent on microbial composition and enzymatic activity. Consequently, the ultimate effect of an excipient may reflect the combined influence of its direct physicochemical properties and its interaction with the intestinal microbiome (Tsunoda et al., 2021).

### 3.2 Surfactants and solubilizers

Surfactants and solubilizers are central to oral formulation development because they can improve wetting, dissolution, solubilization, permeability, and lipid-based delivery (Teama et al., 2025). However, their amphiphilic nature also makes them biologically active at interfaces. A review on oral surfactant excipients emphasized that surfactants can affect epithelial viability and barrier integrity depending on their type and exposure conditions (Maher et al., 2023). This matters for microbiome-aware design because barrier effects and microbiome effects should not be treated separately. A surfactant-rich formulation that perturbs epithelial integrity may also change the luminal environment, mucus interface, or local inflammatory state that shapes microbial ecology (Maher et al., 2023).

Evidence from emulsifier studies supports compound-specific interpretation rather than a class-wide conclusion. *Ex vivo* experiments with human microbiota showed that commonly used dietary emulsifiers can produce different compositional and functional effects (Naimi et al., 2021). These results were generated under food-additive exposure conditions and should not be generalized to finished pharmaceutical products. In a controlled-feeding study, healthy adults received 15 g/day of carboxymethylcellulose for 11 days, with changes in microbiota composition and the fecal metabolome (Chassaing et al., 2022). This is direct evidence from human dietary exposure, but it does not establish equivalent effects at conventional pharmaceutical doses.

For oral dosage-form design, therefore, surfactants and solubilizers should be evaluated according to surfactant load, duration of exposure, patient population, and therapeutic context (Kim K.-S. et al., 2025). The practical design rule is to use the minimum effective surfactant concentration and consider microbiome and epithelial barrier endpoints when the formulation contains high surfactant loads, is intended for chronic administration, or targets patients with intestinal disease. This is especially relevant for self-emulsifying systems, nanoemulsions, and oral nanocarriers that may rely on substantial surfactants or co-surfactant content (Maher et al., 2023).

### 3.3 Polyethylene glycol and related excipients

Polyethylene glycol-related excipients illustrate why dose, grade, molecular weight, and exposure context matter. PEG400 is widely used as a pharmaceutical excipient, but animal evidence suggests that it may alter gut microbiota and fecal metabolomic profiles under specific exposure conditions (Zhao M. et al., 2023). Another mouse study reported that oral PEG400 changed gut microbiota composition in a diet-induced obesity model (Ishibashi et al., 2023). These findings support the view that PEG400 should not be assumed to be microbiome-neutral under all conditions.

In addition, PEG-related excipients should not be treated as a single uniform class. PEG400, macrogols, and higher-molecular-weight PEGs differ in absorption, osmotic activity, formulation function, and expected intestinal exposure (Christoforou et al., 2025). The available evidence is also largely animal-based and often involves exposures that may not directly match typical human pharmaceutical use. Therefore, the appropriate conclusion is to be cautious: PEG-related excipients may be microbiome-active under certain dose, grade, and exposure conditions, but current evidence does not justify labeling all PEG excipients as microbiome-disruptive. In a microbiome-aware framework, PEG-related materials should be considered context-dependent excipients that may require additional testing when exposure is high, repeated, or clinically sensitive (Subramaniam et al., 2023a; Zhao Q. et al., 2023).

### 3.4 Polysaccharide excipients: chitosan, pectin, alginate, dextran, and related materials

Polysaccharide excipients represent a distinct category because their interaction with the microbiome can be an opportunity rather than only a risk. Natural polysaccharides are widely used in oral delivery because they can form matrices, coatings, hydrogels, nanoparticles, and mucoadhesive systems (Musa et al., 2025). A review of microbiota-sensitive polysaccharide systems described natural polysaccharides as materials that can exploit microbial degradation for colon-targeted delivery (Azehaf et al., 2023). This makes polysaccharides important candidates for the proposed category of microbiota-degradable excipients.

The formulation logic is clear. If polysaccharide remains sufficiently intact in the upper gastrointestinal tract but is degraded by enzymes produced by colonic microbiota, it can function as a microbial trigger for site-specific release. Older colon-targeting literature also supports using biodegradable polysaccharides for site-specific colonic drug delivery based on bacterial degradation (Musa et al., 2025). Materials such as pectin, alginate, chitosan, dextran, guar gum, and inulin can therefore be discussed not merely as natural polymers but as microbiome-facing formulation materials (Shuaibu et al., 2026).

The limitation is that microbiota-degradable does not automatically mean predictable. Polysaccharide degradation depends on polymer structure, degree of modification, crosslinking, coating, intestinal transit, disease state, and microbial enzyme abundance. In patients with dysbiosis, inflammatory bowel disease, recent antibiotic exposure, or altered transit, microbial degradation may differ from healthy reference conditions. Therefore, polysaccharide-based systems should be designed with release testing in multi-donor microbiota models and, where needed, combined with pH- or time-dependent protection to reduce premature upper-GI release (Azehaf et al., 2023; Ibrahim, 2023).

Recent formulation studies illustrate both the potential and the translational limitations of this approach. A pectin-chitosan hydrogel containing macrophage-targeted curcumin-loaded liposomes exhibited sensitivity to colonic enzymes during *in vitro* release testing and improved therapeutic outcomes in a dextran sulfate sodium-induced mouse model of ulcerative colitis (Wu et al., 2023). This supports using polysaccharides as protective matrices and microbiota-responsive release components, but the evidence remains preclinical and does not establish predictable performance across human microbiomes.

Conversely, a recent evaluation of pectin-ethylcellulose-coated theophylline pellets found microbially enhanced release in enzyme- and bacteria-containing *in vitro* models, but the effect was not reproduced in healthy or microbiota-depleted pigs. The discordance was associated with low pectinase activity in porcine fecal samples (Giakoumis et al., 2026). This study provides direct evidence that incorporating a microbially degradable polysaccharide is insufficient to guarantee microbiota-responsive release *in vivo*.

Accordingly, classification of a polysaccharide as microbiota-degradable should require evidence that microbial or fecal exposure changes polymer degradation or drug release relative to enzyme-free controls. Conventional dissolution, pH responsiveness, or evidence that the same material is fermentable when consumed as food should be considered indirect. Candidate systems should be evaluated with multiple donor microbiomes, clinically relevant exposure conditions, and disease-relevant samples when the target population includes patients with inflammatory or dysbiotic conditions.

### 3.5 Cellulose derivatives and pH-dependent polymers

Cellulose derivatives and pH-dependent polymers require careful separation because they are often grouped under colon-targeted oral delivery, even though their microbiome relevance differs. Carboxymethylcellulose has relatively strong microbiome-related evidence from human controlled-feeding work, where CMC exposure perturbed microbiota composition and metabolomic profiles (Chassaing et al., 2022). This makes CMC a useful example of a cellulose-derived material that may be microbiome-active or barrier-relevant under certain exposure conditions.

The controlled-feeding evidence for carboxymethylcellulose was generated in healthy adults who received 15 g/day as a dietary emulsifier for 11 days. The intervention altered microbiota composition and fecal metabolomic profiles and reduced diversity in some participants. This is direct human evidence under dietary controlled-exposure conditions, but it should not be interpreted as proof that the smaller quantities used in conventional pharmaceutical formulations produce the same effects.

Hydroxypropyl methylcellulose should not be assigned the same evidence profile as carboxymethylcellulose. In mice, dietary supplementation at 10% of a high-fat diet altered intestinal microbiota composition (Cox et al., 2013). A more recent Caco-2 study reported preservation of epithelial barrier integrity and reduced inflammatory signaling under cytokine exposure (Kim M. et al., 2026). The latter is barrier-related *in vitro* evidence rather than evidence of microbiome modification, and direct human microbiome studies at pharmaceutical exposure levels remain lacking.

pH-dependent polymers, including Eudragit-type materials, have a different role. Their primary function is to control hydration, swelling, dissolution, or drug release according to gastrointestinal pH rather than to undergo microbial degradation. Pectin–Eudragit complexes have been evaluated for physicochemical properties, swelling, and drug release behavior, but those experiments did not demonstrate microbial degradation or a direct effect of Eudragit on microbiota (Nasibullin et al., 2023). Such systems remain relevant to microbiome-aware formulation because the pH-sensitive component can determine where the formulation first encounters dense microbial communities, while the polysaccharide component may provide microbial responsiveness; evidence for each mechanism should be attributed separately.

In the proposed excipient classification, carboxymethylcellulose is, therefore, categorized as having human microbiome evidence under dietary controlled-exposure conditions, with uncertain applicability to pharmaceutical doses. Hydroxypropyl methylcellulose is categorized as having limited and context-dependent microbiome evidence, with separate preclinical evidence relating to barrier effects. pH-dependent polymers are categorized according to their established formulation role and should not be labeled microbiome-active without direct supporting evidence.

By contrast, many pH-dependent polymers are primarily formulation-trigger materials rather than microbiota-degradable materials. Eudragit-type polymers and related pH-sensitive coatings are commonly used to delay release until a target intestinal pH is reached (Lee et al., 2020). Their role in a microbiome-aware framework therefore differs from polysaccharides. They may not directly exploit microbial degradation, but they can control where and when a drug or carrier first encounters dense microbial communities (Gan et al., 2022). This distinction is important because a pH-responsive coating may protect a microbiome-sensitive drug from early microbial exposure, while a microbiota-degradable polymer may intentionally use microbes to trigger release.

The design implication is that selection of pH-dependent and microbiota-dependent strategies should be based on the formulation objective. If the goal is protection from microbial degradation, pH-dependent or enteric protection may be useful. If the goal is colon-specific release, microbiota-degradable or multi-trigger systems may be preferable. If pH variability or disease-associated microbiome variability is expected, combined pH-, time-, and microbiota-responsive designs may offer more robust performance than either trigger alone (Bansal et al., 2026).

### 3.6 Sweeteners, preservatives, and colorants

Unless explicitly identified as pharmaceutical evidence, the studies discussed in this subsection derive from dietary, food-additive, animal, or *in vitro* exposure and should be interpreted accordingly.

Sweeteners, preservatives, and colorants are often present in oral liquids, chewable tablets, pediatric medicines, and patient-friendly dosage forms. Their microbiome relevance is difficult to generalize because much of the evidence comes from food-additive studies rather than pharmaceutical-dose studies. Artificial sweeteners have been reported to alter gut microbiota and glucose tolerance in experimental and human pilot settings (Suez et al., 2014; Hetta et al., 2025). This evidence is important but should be interpreted cautiously in pharmaceutics because medicine exposure patterns, doses, matrices, and patient populations may differ from dietary sweetener exposure.

Colorants provide a more directly formulation-relevant example because some food and drug additives can affect intestinal drug transport (Zou et al., 2020). In that study, bacterial metabolism could reduce the transporter-inhibitory effect of some azo dye additives (Zou et al., 2020). This supports an important nuance: the microbiome is not always only a source of risk. In some cases, microbial metabolism may reduce the biological activity of an excipient or additive.

Preservatives are more difficult to classify because their antimicrobial purpose suggests potential microbiome relevance, but pharmaceutical-dose evidence remains limited. Therefore, preservatives should be discussed as a category needing targeted evaluation rather than as an established microbiome-disruptive class (Schell et al., 2025).

### 3.7 Toward an excipient–microbiome-risk classification

The categories below are presented as the authors’ proposed, evidence-informed framework for formulation research. They are not established regulatory, clinical, or safety classifications, and assignment should remain conditional on the tested dose, exposure duration, formulation function, study model, and patient context.

Category assignment is based on five criteria: directness of the evidence, relevance of the tested exposure to pharmaceutical use, consistency across studies or donors, demonstration of a microbial or barrier consequence, and the importance of that consequence for formulation performance or patient safety. The classification is exposure and context dependent. Accordingly, “microbiome-compatible” denotes no detected concern under the tested conditions rather than proven universal neutrality. In practice, the framework can be used to compare candidate excipients, flag high-load or chronic-use formulations for additional testing, and identify materials whose microbiota-degradability is an intended release function.

The evidence supports a practical excipient–microbiome-risk classification rather than a simple list of excipient effects. The first category is microbiome-compatible excipients, meaning materials with minimal microbiome or barrier effects under tested conditions. The second is microbiome-active excipients, such as PEG400 under certain exposure conditions, which may alter microbiota or metabolites without necessarily being harmful (Zhao M. et al., 2023). The third category includes microbiota-degradable excipients, such as selected polysaccharides that can be used as microbial release triggers (Azehaf et al., 2023). The fourth is microbiome-disruptive or barrier-active excipients, where evidence suggests dysbiosis, reduced diversity, barrier perturbation, or inflammatory potential, such as carboxymethylcellulose (CMC) under controlled dietary exposure (Chassaing et al., 2022). The fifth is microbiome-supportive excipients, a more exploratory category that may include materials such as cyclodextrins when evidence suggests beneficial microbial or metabolite shifts (Varut et al., 2025). A sixth useful category is microbiome-rescued excipients, where microbial metabolism reduces an otherwise relevant excipient or additive effect, as shown for some azo dye-related transport inhibition (Zou et al., 2020).

Overall, excipient–microbiome interactions should be considered within the context of formulation design rather than viewed as universally beneficial or not. The key contribution of this perspective is not simply the recognition that excipients can affect the gut microbiome, but the development of a formulation-centered framework that identifies which excipients warrant microbiome-focused evaluation, which may be exploited in microbiota-responsive delivery systems, and which require caution in chronic-use or vulnerable-patient oral formulations (Morita et al., 2026). Figure 2 illustrates the risk map of different excipients. Table 2 summarizes the excipient classes and reported microbiome-related effects.

**FIGURE 2:**
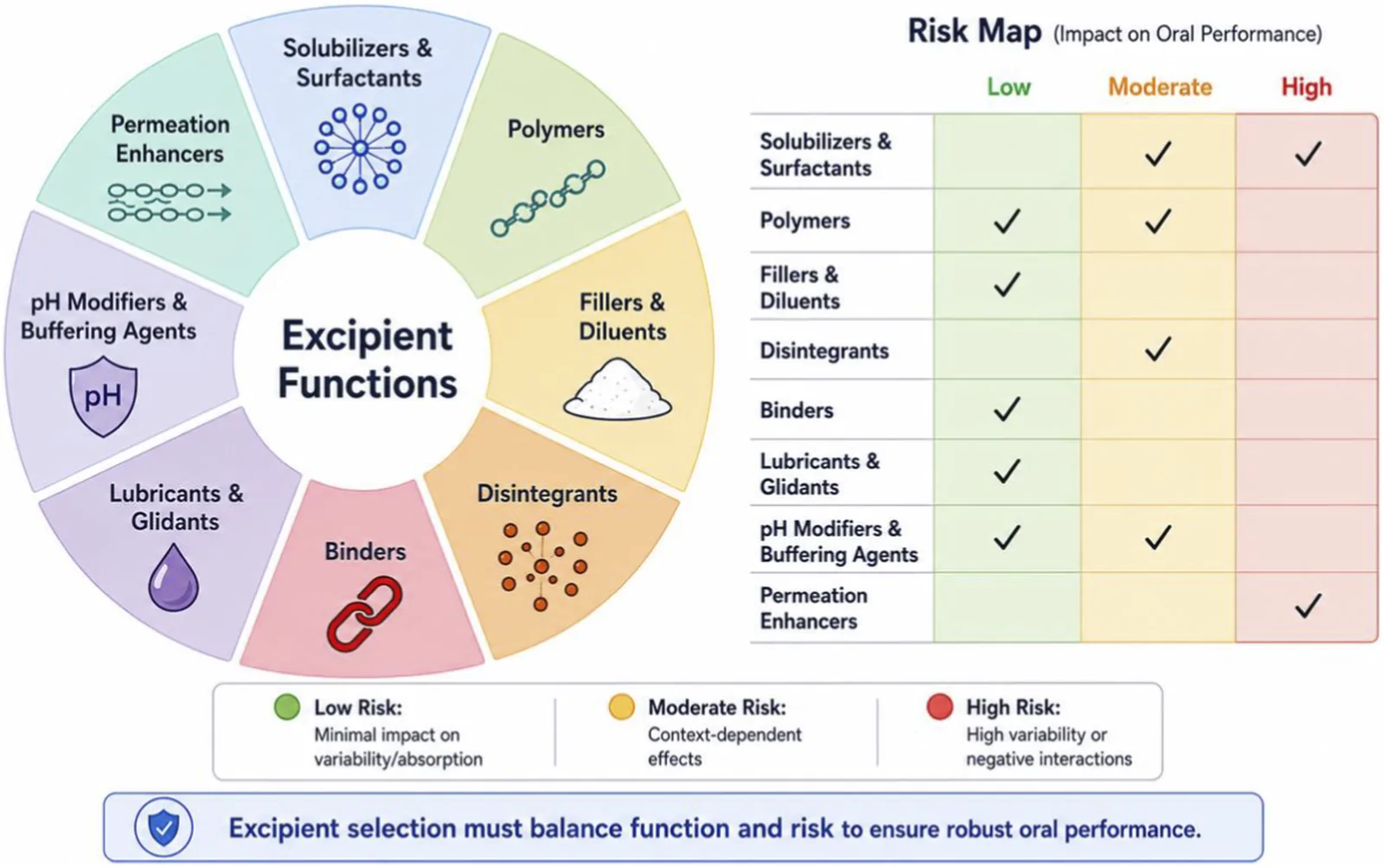
Excipient functional classification and risk map. The figure organizes common excipient functions and maps their potential impact on oral performance into low, moderate, and high-risk categories. The classification supports risk-based excipient selection rather than assuming that all excipients are microbiome-neutral or harmful. This is the authors’ perspective framework and should not be interpreted as an established regulatory or safety classification.

**TABLE 2:** Excipient classes and reported microbiome-related effects.

Excipient class	Examples	Reported microbiome/barrier effect	Formulation use	Potential risk/opportunity	Evidence type, strength, and pharmaceutical applicability	Key reference (s)
Surfactants and solubilizers	Polysorbate 80, Tween, sodium lauryl sulfate (SLS), Cremophor-like agents	Barrier integrity effects; microbiota shifts in some models	Solubilization, permeation enhancement, emulsification	Risk if high load/chronic exposure; opportunity if minimized or replaced with safer alternatives	Predominantly *ex vivo* human-microbiota, animal, and dietary-emulsifier evidence; limited direct human pharmaceutical-dose evidence	Maher et al. (2023), Naimi et al. (2021), Subramaniam et al. (2023a)
Carboxymethylcellulose	CMC	Microbiota and metabolomic perturbation reported during controlled dietary exposure	Binder, viscosity modifier, stabilizer	Potential microbiome-active or barrier-relevant excipient under high or repeated exposure	Human dietary controlled-feeding evidence: relatively strong for 15 g/day food-additive exposure; applicability to ordinary pharmaceutical doses remains uncertain	Chassaing et al. (2022), Naimi et al. (2021)
Hydroxypropyl methylcellulose	HPMC (hypromellose)	Dietary animal microbiota changes; barrier-preserving effects in an inflammatory Caco-2 model	Binder, film former, capsule material, release-controlling matrix	Context dependent; should not be assigned the same evidence profile as CMC	Animal dietary and *in vitro* barrier evidence; low direct evidence for microbiome effects at pharmaceutical exposure	Cox et al. (2013), Kim M. et al. (2026)
PEG-related excipients	PEG400, macrogols	PEG400 altered microbiota/metabolome in mice; effects likely grade/dose/context dependent	Solvent, solubilizer, plasticizer, laxative class at high MW	Microbiome-active under certain exposure settings; avoid overgeneralizing across PEG grades	Moderate	Zhao M. et al. (2023), Ishibashi et al. (2023)
Microbiota-degradable polysaccharides	Pectin, chitosan, alginate, dextran, guar gum, inulin	Microbial or colonic-enzyme exposure may increase matrix degradation, permeability, or drug release	Colon-targeted matrices, coatings, hydrogels, microspheres, and nanoparticles	Opportunity for microbiota-responsive release; risk of incomplete or variable release	Preclinical formulation evidence: *in vitro* enzyme or fecal models and animal studies; strong mechanistic rationale, variable *in vivo* translation, and limited human clinical validation	Azehaf et al. (2023), Jain et al. (2007), Wu et al. (2023), Giakoumis et al. (2026)
pH-dependent polymers	Eudragit-type polymers and enteric coatings	Primarily regulate release location through pH-dependent hydration or dissolution; no established direct microbiome effect	Enteric protection and colonic release	Opportunity to control the site of microbial exposure or form part of a multi-trigger system	Formulation and material evidence: strong for pH-controlled release; low or absent direct evidence of microbiome modification or microbial degradation	Lee et al. (2020), (Rabeh et al., 2024), Nasibullin et al. (2023)
Sweeteners	Saccharin, sucralose, aspartame	Microbiome effects reported but human evidence is mixed and dose dependent	Palatability in oral liquids/chewables	Use with caution in chronic/vulnerable populations; pharmaceutical-dose validation is needed	Primarily dietary, animal, and human pilot evidence; direct pharmaceutical-dose evidence is limited and mixed	Suez et al. (2014), YLee et al. (2021)
Preservatives and colorants	Sodium benzoate, nitrite, sorbate, FD&C azo dyes	Antimicrobial or transporter effects; bacterial metabolism may reduce some azo dye effects	Preservation and appearance	Potential microbiome-active/rescued excipients; use claim cautiously	Mainly *in vitro*, food-additive, and transporter evidence; pharmaceutical microbiome effects require compound- and dose-specific confirmation	Zou et al. (2020), Li et al. (2022), Davoudi et al. (2021)
Cyclodextrins and supportive candidates	alpha-, beta-, and gamma-cyclodextrins	May support beneficial bacteria or short-chain fatty acid (SCFA) production depending on type/dose	Solubilization and inclusion complexes	Potential microbiome-supportive or active excipient class; needs compound-specific testing	Moderate	Varut et al. (2025)

## 4 Nanocarrier–microbiome interface

Oral nanocarriers are commonly designed to improve drug solubility, protect labile molecules, enhance permeability, control release, increase local retention, or target specific gastrointestinal regions. In conventional formulation development, their performance is often evaluated using particle size, polydispersity index, zeta potential, encapsulation efficiency, release kinetics, stability in simulated gastrointestinal media, permeability, and pharmacokinetic outcomes (Hocaoğlu et al., 2026). A microbiome-aware perspective does not replace these classical parameters; it adds another interface to consider when the nanocarrier encounters mucus, luminal microbes, microbial enzymes, bile acids, epithelial barriers, and microbial metabolites. This is especially relevant for oral nanocarriers intended for chronic use, local intestinal therapy, colon targeting, or delivery systems with high excipient and surfactant loads (Parodi et al., 2021).

### 4.1 Why oral nanocarriers need microbiome-aware engineering

Nanocarriers should not be treated as microbiome-neutral by default. A systematic review of nanomaterial effects on gut microbiota reported that microbiota-related outcomes can depend on size, dose, exposure duration, and functional groups (Utembe et al., 2022). These variables overlap strongly with formulation design parameters. Particle size not only determines stability and uptake; it may also influence residence time, mucus diffusion, and microbial exposure. Surface functional groups are not only tools for targeting or stabilization; they may also affect interactions with bacterial membranes, mucus components, and epithelial surfaces. Dose and exposure duration are also critical because a single acute dose may have different microbiome relevance from repeated administration of a chronic oral nanomedicine (Maisel et al., 2015; Utembe et al., 2022).

Oral nanomedicine literature also supports considering the intestinal barrier and gut microbiome together. Oral nanocarriers have been shown to modulate intestinal barrier function and gut microbiota composition, with effects documented across both disease and healthy exposure models (Lee et al., 2021). However, most oral nanocarrier studies still prioritize delivery efficiency, drug loading, and therapeutic outcomes over microbiome safety or microbiome-mediated variability. This creates a translational gap: a formulation may improve bioavailability or local delivery in a conventional model, yet its repeated interaction with the microbiome, mucus, and barrier may remain poorly characterized (Zheng et al., 2025). Therefore, oral nanocarrier engineering should include an evaluation of microbiome-facing layers whenever the formulation is likely to have prolonged luminal exposure, local intestinal action, or microbiota-responsive behavior (Gelli et al., 2025).

### 4.2 Polymeric nanoparticles

Polymeric nanoparticles are highly relevant to microbiome-aware oral delivery because their material composition can influence degradation, mucoadhesion, surface charge, release behavior, and intestinal interaction. Poly (lactic-co-glycolic acid) (PLGA)-based nanoparticles, chitosan-coated systems, alginate-coated systems, and other polymeric carriers are widely investigated for oral and intestinal delivery because they can protect drugs and modulate release. Chitosan and alginate nanoparticles are particularly important because they combine biodegradable polymeric delivery with mucus and epithelial interactions (Li et al., 2022). Chitosan is often used to increase mucoadhesion and improve epithelial contact, while alginate can provide a negatively charged, gel-forming, and pH-responsive material environment (Amin and Boateng, 2022).

Surface coating studies show that polymeric nanoparticles can be engineered to change intestinal interaction patterns. In intestinal organoid models, alginate and chitosan coatings have been used to impose different surface charges on PLGA nanoparticles and examine how charge affects delivery behavior (Davoudi et al., 2021). This is formulation-relevant because surface charge can influence mucus binding, epithelial interaction, particle aggregation, and possibly microbial contact. A positively charged particle may improve adhesion or epithelial interaction, but it may also be trapped in mucus or interact more strongly with microbial surfaces. A negatively charged or neutral coating may reduce nonspecific binding and improve diffusion, but it may reduce local retention. Therefore, charge should be selected according to the intended delivery goal rather than optimized in a single direction (Porfiryeva et al., 2026).

Polysaccharide-containing polymeric nanoparticles add another microbiome-specific dimension. Natural polysaccharides can be degraded by enzymes from colonic microbiota and used in microbiota-sensitive colon-targeted systems (Azehaf et al., 2023). When incorporated into nanoparticles or used to coat natural materials, they may improve particle stability, release control, and microbial-triggered degradation. This makes polysaccharide-based nanoparticles a bridge between conventional nanocarrier design and microbiota-responsive oral delivery. However, microbial degradation depends on patient microbiome function, disease activity, antibiotic exposure, and polymer modification. Thus, polymeric nanoparticles designed to exploit microbial degradation should be tested across multiple microbiome conditions rather than only in standard simulated fluids (Campos et al., 2022).

Representative preclinical studies show why polymer identity and surface behavior must be interpreted together with microbiome outcomes. In a mouse model of dextran sulfate sodium-induced colitis, orally administered low-molecular-weight water-soluble chitosan nanoparticles carrying covalently attached bilirubin showed prolonged gastrointestinal retention attributed to chitosan mucoadhesion, improved intestinal barrier and mucosal immune readouts, and attenuated expansion of the inflammation-associated genus Turicibacter (Rahman et al., 2023). This provides direct animal evidence linking a polymeric nanocarrier to therapeutic and microbiome outcomes, but it does not isolate whether the microbiome change resulted from chitosan, bilirubin, improvement of inflammation, or their combination.

Accordingly, polymeric nanocarrier studies should include blank-carrier and free-drug controls, characterize luminal exposure and polymer degradation, and determine whether microbiome shifts preceded or followed disease improvement. In the absence of these controls, restoration of microbiota composition should be interpreted as an associated treatment outcome rather than proof that the carrier directly modulates the microbiome.

### 4.3 Lipid-based nanocarriers: liposomes, solid lipid nanoparticles, and nanostructured lipid carriers

Lipid-based nanocarriers, including liposomes, solid lipid nanoparticles, nanostructured lipid carriers, and related lipid systems, are attractive for oral delivery because they can improve solubilization, protect drugs, and support local or systemic delivery (Plaza-Oliver et al., 2021). In the gastrointestinal tract, however, lipid systems encounter bile salts, digestive enzymes, dietary lipids, mucus, and microbial metabolites. Reviews of oral lipid nanoparticles for intestinal disease highlight the importance of composition, surface functionalization, and coating in improving gastrointestinal delivery (Zhu et al., 2025). These same design variables may also influence how the lipid carrier interacts with the microbiome-containing intestinal environment.

The microbiome-specific evidence for pharmaceutical oral lipid nanoparticles is less developed than for some polysaccharide systems or surfactant-rich self-emulsifying systems (Plaza-Oliver et al., 2021). Lipid nanocarriers are not automatically microbiome-disruptive, but their excipient composition, digestion products, surfactants, and coatings may alter luminal exposure and barrier interaction. Bile acid metabolism is also relevant because the gut microbiome modifies bile acids, and bile acid pools can influence lipid digestion, micellar solubilization, and intestinal absorption (Wahlström et al., 2016). Therefore, microbiome-aware design of lipid nanocarriers should not focus only on particle stability and drug release but also on lipid composition, surfactant burden, bile interaction, digestion products, and repeated exposure.

For practical formulation design, lipid nanocarriers should be evaluated according to the intended site of action. For systemic absorption, the key question is whether the lipid system improves solubilization and epithelial transport without causing undesirable barriers or microbiome effects (Woo et al., 2025). For local microbiome therapy, the main question is whether the carrier remains stable long enough to reach the target region and whether its excipients alter the inflamed or dysbiotic local environment. This distinction prevents overgeneralization and keeps the microbiome discussion connected to formulation purpose (Yaqub et al., 2025).

A comparative 6-gingerol study illustrates that lipid carrier architecture can change both delivery location and microbiome-associated outcomes. The nanostructured lipid carrier was predominantly absorbed in the small intestine, whereas incorporation of the same carrier into a microcapsule shifted accumulation toward colonic lesions and produced stronger therapeutic effects accompanied by better preservation of the mucus barrier, microbial structure and diversity, and intestinal barrier function (Tian et al., 2022). Because drug distribution and carrier format changed simultaneously, the study supports design-level relevance but does not establish a direct causal effect of the lipid particles on specific bacterial taxa.

An oral plant-lipid-inspired nanoparticle carrying interleukin-22 mRNA provides a complementary example. The formulation increased interleukin-22 expression in colonic mucosa and accelerated healing in experimental colitis (Sung et al., 2022). The study demonstrated therapeutic efficacy but did not establish whether the benefit involved microbiome remodeling. Therapeutic efficacy of an oral lipid nanocarrier should not be interpreted as direct microbiome modulation unless microbiome endpoints are measured and reported.

Recent vesicular platforms also illustrate how conventional oral nanocarrier optimization remains centered on biopharmaceutical performance. Hybrid niosomes improved apixaban oral exposure and anticoagulant pharmacodynamics (Ragheb et al., 2026), whereas Cremophor RH 40-containing cremosomes increased ticagrelor oral bioavailability by approximately 344% relative to drug suspension through combined solubilization and P-glycoprotein modulation (AbouHussein et al., 2026). These studies provide recent formulation performance examples but did not include gut microbiome endpoints. They therefore support extending the evaluation of surfactant-rich oral nanocarriers beyond physicochemical and pharmacokinetic outcomes when repeated luminal exposure makes a microbiome-facing effect plausible.

### 4.4 Nanoemulsions and self-emulsifying systems

Nanoemulsions and self-emulsifying systems are among the most important links between excipient science and nanocarrier microbiome safety. Self-emulsifying drug delivery systems (SEDDSs), self-microemulsifying drug delivery systems (SMEDDSs), and self-nanoemulsifying drug delivery systems (SNEDDSs) are widely used to improve oral delivery of poorly water-soluble drugs by combining oils, surfactants, co-surfactants, and solvents to generate fine dispersions in gastrointestinal fluids (Uttreja et al., 2025). Their strength is also their microbiome-relevant concern: the same surfactants and solubilizers that improve dispersion and absorption may interact with epithelial barriers, mucus, and microbial communities during repeated oral exposure.

A rat study reported that repeated oral administration of SEDDSs disrupted gut microbiota and triggered an intestinal inflammatory response (Subramaniam et al., 2023b). This does not prove that all SEDDSs are unsafe in humans, but it provides a formulation-specific warning signal. It supports the need to evaluate surfactant-rich systems beyond conventional dissolution and pharmacokinetic tests, especially when chronic dosing is expected. The design implication is direct: SEDDSs should be optimized for droplet size, drug loading, and bioavailability, as well as for surfactant load, exposure duration, barrier compatibility, and microbiome safety.

One possible design direction is to reduce synthetic surfactant burden or replace part of the emulsification function with lower-risk or functional alternatives (Govindan et al., 2025). A formulation study using lactoferrin to reduce Tween content in SEDDSs illustrates how surfactant reduction can be treated as a formulation objective (Khairnar et al., 2024). Although this type of study does not by itself prove microbiome benefit, it supports a broader design principle: when two formulations provide similar bioavailability, the one with lower microbiome/barrier risk should be preferred, especially for repeated-dose oral therapy (Uttreja et al., 2025).

Direct formulation-specific safety evidence is available for self-emulsifying drug delivery systems. Daily oral administration of representative Type I, Type II, and Type III lipid formulations to rats for 21 days altered gut microbiota composition and diversity, with dysbiosis signatures correlating with jejunal pro-inflammatory cytokine expression and reduced plasma citrulline, a marker of enterocyte mass and mucosal integrity (Subramaniam et al., 2023b). The study provides a repeated-dose preclinical warning signal, but its fixed dose and rat model do not define a universal human toxicity threshold for individual surfactants or commercial self-emulsifying products.

Surfactant minimization can therefore be treated as a measurable formulation objective. In a fenofibrate self-emulsifying system, partial substitution with lactoferrin reduced the combined Tween 80 and Span 80 content by 27% while retaining broadly comparable physicochemical performance and improving oral exposure in rats (Khairnar et al., 2024). Because the study did not assess microbiota or barrier endpoints, it supports the feasibility of lowering synthetic surfactant burden but is not proof of microbiome protection. Candidate low-surfactant systems still require direct comparison with the conventional formulation in microbiome-relevant assays.

### 4.5 Chitosan-, pectin-, and alginate-based nanocarriers

Chitosan-, pectin-, and alginate-based nanocarriers deserve separate attention because they combine several microbiome-facing properties: polymeric structure, charge, mucoadhesion, microbial degradability, and potential colon-targeting utility. Chitosan-based systems can provide positive charge and mucoadhesive behavior, which may improve local retention or epithelial interaction (Li et al., 2022). Alginate and pectin can support gel formation, pH-dependent behavior, and potential degradation by colonic microbiota (Azehaf et al., 2023). These materials therefore function not only as carrier matrices, but also as interface-modifying excipients.

Recent pectin/chitosan nanoparticle systems for ulcerative colitis point to a shift toward responsive, locally acting oral nanocarriers (Wu et al., 2025). These systems are promising because they align material choice with disease location and microbiota-relevant release. However, most remain preclinical and should be evaluated using disease-relevant microbiota, inflammatory barrier models, and release testing that includes fecal or fermentation-based media. Without these tests, a system may appear colon-targeted in simplified media but behave differently in dysbiotic patients (Chigozie et al., 2025).

One recent architecture combined macrophage-targeted curcumin-loaded liposomes with a pectin-chitosan hydrogel. The polysaccharide matrix provided upper gastrointestinal protection and colonic-enzyme-sensitive release, while the liposomal component supported cellular targeting and therapeutic activity in a preclinical ulcerative colitis model (Wu et al., 2023b). This layered design is a useful example of assigning distinct functions to the matrix and the nanoparticle. However, human donor variability and the relative contributions of pH, swelling, and microbial enzymes remain unestablished.

A 2026 study used a different multilayer strategy: macrophage-derived extracellular vesicles carrying 5-aminosalicylic acid were sequentially coated with a cationic lipid and sodium alginate. The coating increased resistance to gastric and enzymatic stress, enabled delayed uncoating at colonic pH, improved epithelial and macrophage delivery, and reduced disease severity in experimental colitis (Cho et al., 2026). The study did not demonstrate microbiota-dependent alginate degradation or profile microbiome effects; it should therefore be interpreted as a pH-protective alginate-coated nanocarrier rather than a validated microbiota-responsive system. This distinction prevents the presence of a natural polysaccharide from being treated as evidence of microbial triggering.

### 4.6 Nanocarrier properties: size, charge, surface chemistry, and mucoadhesion

A microbiome-aware nanocarrier framework should organize design around properties rather than carrier names alone. Size influences diffusion through mucus, interaction with epithelial cells, residence time, and exposure to luminal microbes. Surface charge affects mucus adhesion, aggregation, epithelial contact, and possibly interactions with bacterial membranes (Eldin et al., 2026). Surface chemistry and coating influence protein adsorption, mucus penetration, enzymatic degradation, and stability in gastrointestinal fluids. A systematic review of nanomaterial-microbiota effects identified size, dose, duration, and functional groups as variables that shape microbiota outcomes (Utembe et al., 2022). These variables should therefore be treated as candidate microbiome-facing design attributes.

Mucus interaction is particularly important. Oral nanocarriers must either penetrate mucus, adhere to mucus, or remain in the lumen depending on the intended therapeutic goal. Reviews of mucus-interacting nanoparticles distinguish between mucoadhesive systems that improve local retention and mucus-penetrating systems that improve access to the epithelial surface (Liu et al., 2021). This distinction has direct implications for microbiome-aware design. A system intended for local colonic therapy may benefit from mucoadhesion, whereas a system intended for systemic absorption may require mucus penetration and reduced nonspecific binding. In both cases, microbial and mucus environments should be considered together because bacteria, mucins, and microbial metabolites influence the luminal interface encountered by the nanocarrier (Porfiryeva et al., 2026).

### 4.7 Nanocarrier-induced microbiome disruption and safety concerns

The safety question should be framed carefully. The current evidence does not support a universal claim that oral nanocarriers disrupt the microbiome. However, it does support a risk-based testing approach. Nanomaterials can affect gut microbiota in both *in vivo* and *in vitro* studies, and the direction and magnitude of these effects vary by physicochemical properties and exposure conditions (Utembe et al., 2022). Long-term oral exposure to some dietary nanoparticles has been associated with changes in gut microbiome composition and function in mice (Perez et al., 2021). These are not direct proof for all pharmaceutical nanocarriers, but they are relevant warning signals for repeated oral exposure.

For pharmaceutical development, microbiome safety should be prioritized when the formulation is chronic, acts locally in the gut, or is surfactant-rich, antimicrobial, non-biodegradable, highly cationic, or designed for microbiota-responsive release. Minimum safety endpoints should include microbiome composition, microbial functional capacity, key metabolites such as SCFAs and bile acids, mucus/barrier integrity, inflammation markers, and conventional formulation performance endpoints (Al-Btoosh et al., 2026). The goal is not to add microbiome testing to every oral formulation indiscriminately. The goal is to identify high-risk or high-opportunity formulations where microbiome data can meaningfully improve design decisions.

Overall, oral nanocarriers should be understood as microbiome-interface systems rather than passive delivery vehicles. The strongest microbiome-aware design message is that nanocarrier properties should be selected according to the intended interaction with the gastrointestinal ecosystem: protect from microbial metabolism, exploit microbial degradation, penetrate mucus, adhere locally, minimize barrier disruption, or reduce repeated-exposure microbiome risk. This property-based logic provides the foundation for microbiota-responsive delivery systems discussed in the next section. Figure 3 summarizes the nano-strategies to overcome oral delivery barriers. Table 3 summarizes the oral nanocarriers interacting with or exploiting the gut microbiome.

**FIGURE 3:**
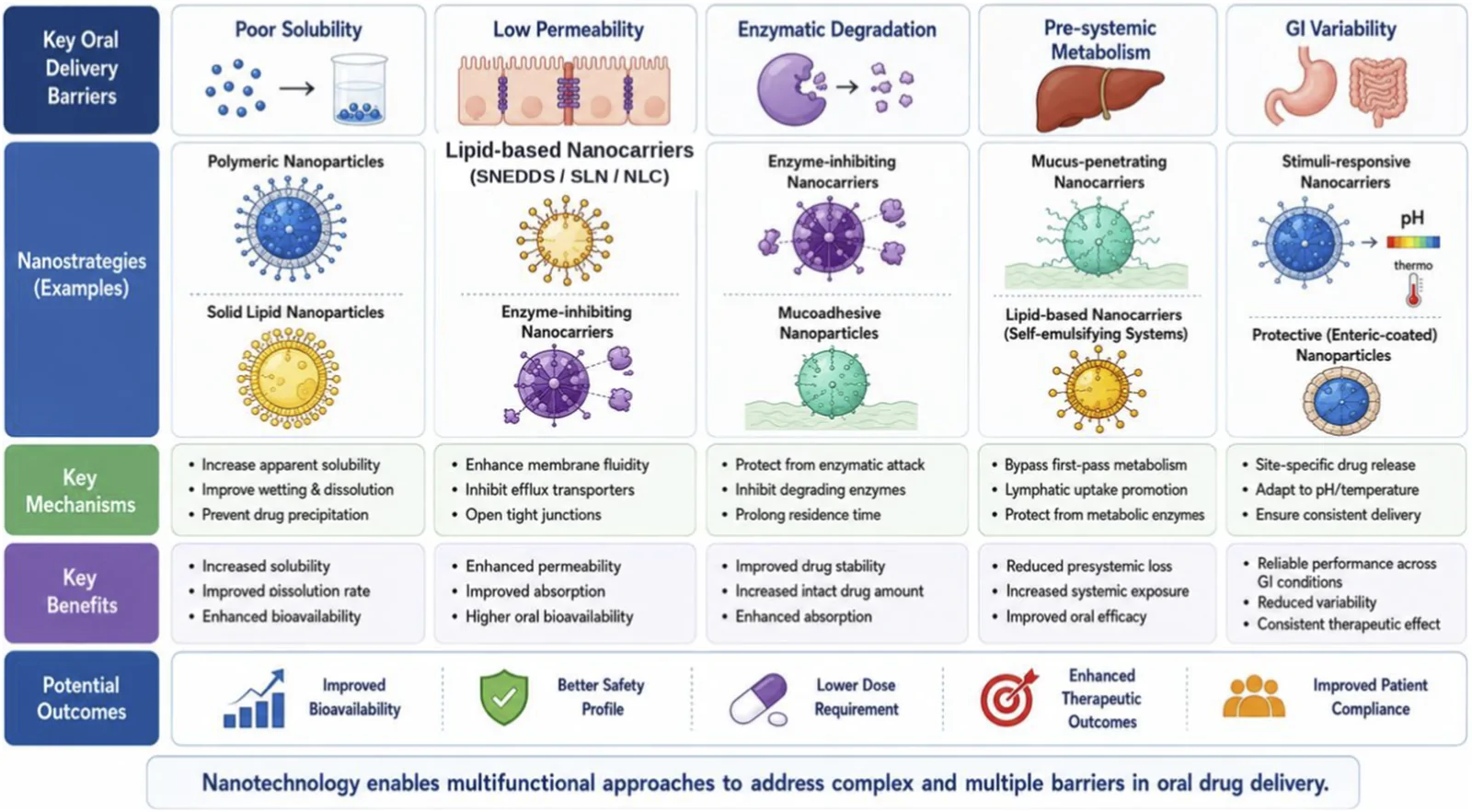
Nano-strategies to overcome oral delivery barriers. The schematic links major oral delivery barriers, including poor solubility, low permeability, enzymatic degradation, presystemic metabolism, and gastrointestinal variability, representative nanocarrier strategies, mechanisms, benefits, and expected formulation outcomes.

**TABLE 3:** Oral nanocarriers interacting with or exploiting the gut microbiome.

Nanocarrier type	Material/composition	Microbiome interface	Design opportunity	Main concern	Evidence strength	Key reference (s)
Polymeric nanoparticles	PLGA, chitosan-coated PLGA, alginate-coated PLGA	Mucus, epithelium, possible microbial interface; surface coating controls charge	Controlled release; surface charge tuning; protection of payload	Microbiome endpoints often absent; charge may increase mucus binding	Moderate	Subramaniam et al. (2023b), Liu et al. (2021)
Polysaccharide nanocarriers	Chitosan, pectin, alginate, dextran, inulin systems	Microbial enzyme degradation and colon microbiota exposure	Microbiota-responsive colon release; local therapy	Release reliability depends on enzyme abundance and dysbiosis	High for mechanism; clinical validation variable	Azehaf et al. (2023)
Lipid nanoparticles/SLNs/NLCs	Lipid matrices, coated SLNs/NLCs	Bile, digestion, mucus, microbiome exposure	Improve local GI or colonic targeting; protect drug	Direct microbiome-specific evidence is less developed	Moderate	Zhu et al. (2025), Zhang et al. (2023)
Liposomes	Phospholipid vesicles	Mucus/barrier interface; digestion/bile exposure	Local intestinal delivery; encapsulation of labile drugs	GI stability and microbiome interaction data limited	Moderate	Rekdal et al. (2019), Zhu et al. (2025)
Nanoemulsions/SEDDS	Oils + surfactants + co-surfactants	Surfactant-microbiome/barrier interface	Improve oral bioavailability of poorly soluble drugs	Repeated-dose microbiome/barrier disruption risk in rats; composition-specific	High for SEDDS risk signal	Subramaniam et al. (2023b)
Mucus-penetrating nanoparticles	Neutral/hydrophilic shields, PEG-like coatings	Mucus mesh penetration and epithelial access	Systemic absorption or epithelial delivery	May reduce local retention; microbiome effect indirect	Moderate-high	Liu et al. (2021)
Mucoadhesive nanoparticles	Chitosan/cationic/adhesive polymers	Mucus retention; possible bacterial/membrane interactions	Local GI/colon therapy and prolonged residence	Mucus trapping, barrier irritation, or charge-related safety concerns	Moderate-high	Subramanian et al. (2022), Liu et al. (2021)
Microbial-enzyme-responsive nanocarriers	Azo-linked micelles, cellulase-responsive SLNs, enzyme-responsive hydrogels	Specific bacterial enzymes trigger release	Programmable colonic release and local therapy	Patient enzyme variability and preclinical evidence	High for proof-of-concept; translation pending	Tang et al. (2024), Zhang et al. (2023), Rabeh et al. (2024)

## 5 Microbiota-responsive oral drug delivery systems

Microbiota-responsive oral drug delivery systems convert the gut microbiome from a source of variability into a potential formulation trigger. The main idea is that selected microbial enzymes, reducing conditions, or microbial degradation pathways can be used to release drugs at intestinal or colonic sites (Zhang et al., 2021). This strategy is especially attractive for colon-targeted therapy because the colon contains dense microbial communities and enzyme activities that differ from the upper gastrointestinal tract. However, microbiota-responsive delivery is not automatically predictable. Its performance depends on whether the microbial trigger is sufficiently localized, sufficiently abundant, and sufficiently stable across patients, disease states, diet, antibiotic exposure, and intestinal transit conditions (Philip and Philip, 2010).

### 5.1 Colon-targeted systems based on microbial enzymes

Colon-targeted oral delivery aims to protect the drug during passage through the stomach and small intestine, then release it at or near the colon. Microbiota-sensitive polysaccharide systems are widely discussed for this purpose because they can use microbial degradation in the distal gut as a site-specific release mechanism (Azehaf et al., 2023). Colon-targeted oral delivery systems also include pH-dependent, time-dependent, pressure-dependent, and enzyme-dependent strategies, each with different advantages and failure modes (Amidon et al., 2015). From a microbiome-aware formulation perspective, microbial enzyme-triggered systems are particularly interesting because the trigger is endogenous to the target region rather than externally applied (Chourasia and Jain, 2003).

The value of microbial enzymes in a formulation is that they can provide biological specificity. A polymer, prodrug, hydrogel, coating, or nanoparticle can be designed to remain relatively stable in upper gastrointestinal conditions and degrade or cleave when exposed to colonic microbial enzymes (Zhou et al., 2026). Dual pH- and enzyme-responsive delivery systems show how microbial triggers can be combined with classical formulation cues to improve site-specificity (Rabeh et al., 2024). This combined-trigger logic is important because pH-only systems can fail when gastrointestinal pH varies, while microbiota-only systems can fail when microbial activity is altered (Friend and Chang, 1984). Therefore, colon-targeted design should consider not only where the drug should be released, but which trigger or trigger combination is most reliable for the intended patient population (Utembe et al., 2022).

### 5.2 Polysaccharide degradation as a release trigger

Polysaccharide degradation is one of the most important microbiota-responsive release strategies. Natural polysaccharides can serve as substrates for microbial enzymes in the distal gut and are therefore attractive materials for colon-targeted delivery (Azehaf et al., 2023). Materials such as polysaccharides can be used as matrices, coatings, nanoparticles, or hydrogel components. Their value is that they are both biocompatible formulation materials and that their degradation can be linked to colonic microbial activity (Musa et al., 2025).

Older colon-delivery literature provides the mechanistic foundation for using biodegradable natural polysaccharides in site-specific delivery to the colon. In modern formulation design, this principle can be extended into composite systems that combine polysaccharide degradation with pH-sensitive coatings, crosslinking, time-dependent erosion, or nanoparticle architecture (Gong et al., 2024). The design challenge is to avoid premature release in the upper gastrointestinal tract while preserving sensitivity to colonic microbial degradation. If the polysaccharide hydrates or degrades too early, the system may release before reaching the target site. If it is too heavily modified or crosslinked, microbial degradation may become insufficient for timely release (Barclay et al., 2019).

For this reason, polysaccharide-triggered systems should be evaluated in microbiome-containing media, not only in standard simulated gastric and intestinal fluids. Release testing using fecal slurry, fermentation models, or dynamic colon systems can help determine whether microbial degradation is strong enough to support the intended release profile. Because microbial polysaccharide-degrading capacity varies between individuals, multi-donor testing is particularly important for systems intended for patients with dysbiosis or inflammatory bowel disease (Gong et al., 2024; Yaqub et al., 2025).

### 5.3 Azo bond and bacterial reduction-based systems

Azo bond-based systems represent a classical microbiota-responsive strategy. Azo bonds can be cleaved by bacterial azoreductases in the colon, enabling drug release from azo prodrugs, azo polymers, or azo-linked delivery systems. This principle is historically important because it demonstrates how a chemical bond can be selected specifically for microbial cleavage. In formulation terms, the azo linkage functions as a microbial reduction-sensitive switch (Pinheiro et al., 2022).

Modern systems continue to adapt this concept. Azoreductase-responsive polymeric prodrugs have been developed for sustained colon-targeted release of 5-aminosalicylic acid in colitis therapy (Tang et al., 2024). This shows that azo chemistry is not only a historical prodrug strategy but can also be incorporated into contemporary polymeric or nano-enabled delivery systems. Dual-stimuli hydrogels further show how bacterial reduction can be paired with pH responsiveness to create multi-triggered colon-targeted release (Rabeh et al., 2024). These designs are formulation-relevant because they reduce dependence on a single environmental cue.

The limitation is that azoreductase activity is microbiome dependent. If bacterial reductase activity is reduced or altered, azo cleavage may become delayed, incomplete, or variable. Therefore, azo-based systems should be tested under microbiome conditions that reflect intended clinical use. This is especially important for inflammatory bowel disease because the target population may have altered microbial composition and enzyme activity. The design rule is to use azo triggers when colonic bacterial reduction is likely to be reliable and to consider multi-trigger safeguards when disease-associated variability is expected (Zahran et al., 2019).

### 5.4 Glycosidase-responsive and enzyme-responsive systems

Glycosidase-responsive systems use another microbial enzyme logic. Drug glycosides were historically proposed as colon-specific delivery systems based on cleavage by glycosidases produced by colonic bacteria. This strategy relies on the fact that certain glycosidic linkages can resist upper gastrointestinal conditions but be cleaved by bacterial enzymes in the colon. Like azo systems, glycosidase-responsive designs translate microbial enzymology into a release mechanism (Overkleeft et al., 2026).

More recent enzyme-responsive systems extend the concept beyond classical glycosides. Microbial enzyme-responsive solid lipid nanoparticles have been designed with cellulase-sensitive layers to enable colonic budesonide release for ulcerative colitis therapy (Zhang et al., 2023). This is important because it shows that microbial enzyme responsiveness can be engineered into nanocarrier surfaces, not only into small-molecule prodrugs or simple polymer matrices. Such systems may allow more precise control over release site, local exposure, and drug protection.

However, enzyme-responsive delivery should be treated as a design opportunity with a built-in variability risk. Enzyme activity can differ between healthy individuals and patients, between inflamed and non-inflamed intestinal regions, and before or after antibiotic exposure (Zhou et al., 2026). For this reason, enzyme-responsive systems should be evaluated using functional assays that measure release under microbiome-relevant conditions, rather than relying only on chemical stability or standard dissolution data (Xu et al., 2026).

### 5.5 Variability of microbiota-responsive release between patients

The central challenge for microbiota-responsive delivery is predictability. Personalized microbiome studies show that human gut microbial communities can differ in their drug-metabolizing capacity (Javdan et al., 2020). Although drug metabolism is not identical to formulation release, the same principle applies: microbial function is not uniform across patients. Therefore, a microbiota-triggered dosage form may not release identically in all individuals (Burke and Li, 2025).

Disease state is a particularly important source of variability. Bacterial enzymes used for colon-specific drug delivery have been reported to decrease in active Crohn’s disease. This finding is directly relevant to colon-targeted systems because many intended users of microbiota-responsive formulations may have inflammatory bowel disease or other forms of intestinal dysbiosis. A delivery system designed using healthy microbiota may therefore perform differently in the actual target population (Gunawan et al., 2025).

This variability does not invalidate microbiota-responsive oral delivery, but it does change how such systems should be designed and tested. Microbiota-responsive formulations should be evaluated across multiple donor microbiomes, disease-relevant microbiota, and conditions that simulate antibiotic exposure or altered microbial enzyme activity where appropriate. When variability is expected, multi-trigger systems combining pH, time, and microbial enzyme sensitivity may perform more robustly than microbiota-only designs (Rabeh et al., 2024).

## 6 Experimental models for microbiome-aware formulation testing

Microbiome-aware oral formulation design requires experimental models that can connect three layers: formulation performance, microbial function, and host-relevant intestinal responses (Pires et al., 2024). Conventional dissolution, stability, permeability, and pharmacokinetic tests remain essential, but they are not sufficient when the formulation is expected to interact with gut microbes, microbial enzymes, mucus, epithelial barriers, or microbiome-derived metabolites. The central testing challenge is that no single model can reproduce all relevant aspects of the gastrointestinal environment. Therefore, microbiome-aware testing should follow a model hierarchy, beginning with controlled microbial screening and progressing toward dynamic, epithelial, organoid, animal, and ultimately patient-relevant validation when needed (Adelfio et al., 2025; Wang et al., 2026).

### 6.1 *In vitro* fermentation models

*In vitro* fermentation models are useful first-line systems for studying how oral formulations, excipients, or nanocarriers interact with gut microbial communities. These models allow culture of complex intestinal microbiota under controlled environmental conditions (Verhoeckx et al., 2015). Their main value is screening: formulation-driven changes in microbial composition, microbial degradability of materials, metabolite profile shifts following exposure, and microbiota-triggered drug release. Batch fermentation is especially useful for early, relatively fast screening across multiple donor microbiomes, while more complex continuous models can better approximate repeated exposure (Adelfio et al., 2025).

However, fermentation models are not full intestinal models. They do not reproduce epithelial absorption, active transport, immune responses, mucus turnover, systemic pharmacokinetics, or host feedback (Adesina et al., 2025). Therefore, a positive or negative fermentation signal should be interpreted as microbial evidence, not as complete proof of formulation safety or performance. Their best use is to identify microbiome-related risks or opportunities early before moving to more complex testing (Adesina et al., 2025; Massinai et al., 2026).

Model design should begin with the specific formulation question. For microbial drug metabolism, the primary endpoint may be loss of parent drug and formation of microbial metabolites; for microbiota-degradable carriers, it may be coating erosion or drug release; and for excipient safety, it may be community composition, growth inhibition, metabolite production, or functional pathway changes. Batch systems should maintain anaerobiosis, temperature, and pH relevant to the intended colonic region and should report inoculum preparation, donor characteristics, medium composition, test-article concentration, dosing basis, incubation time, and whether the formulation underwent simulated gastric and small-intestinal digestion before fermentation.

At minimum, the experimental design should include a fermentation blank, a vehicle or excipient control, free-drug and complete-formulation arms when relevant, and a sterile or microbiota-free control for distinguishing chemical release from microbial transformation. Concentrations should reflect the estimated amount reaching the colon rather than the nominal oral dose. Readouts should pair formulation analytics, including parent drug, metabolites, particle or matrix integrity, and release kinetics, with microbial endpoints such as cell density, 16S ribosomal RNA (16S rRNA) or metagenomic composition, targeted functional genes, short-chain fatty acids, and broader metabolomics. A published batch-fermentation protocol for endobiotic-microbiota interactions demonstrates how compositional and metabolic readouts can be integrated under controlled conditions (Hu et al., 2019).

Donor strategy strongly affects interpretation. Individual-donor fermentations preserve responder and non-responder variation, whereas pooled inocula improve throughput but can conceal low-abundance metabolic phenotypes. An *in vitro* colonic simulator using fecal slurries from five donors showed rapid sulfasalazine reduction in all donors but different degradation rates among related azo prodrugs (Sousa et al., 2014). This supports using several individual donors to confirm the mechanism and a predefined pooling strategy only for later comparative screening.

Formulation studies also need controls that separate microbial triggering from pH-, time-, swelling-, and diffusion-controlled release. In one multi-trigger platform, fecal bacteria from patients with inflammatory bowel disease accelerated release from pectin-containing coatings relative to control culture medium, showing how disease-relevant inocula can reveal a microbial contribution (Moutaharrik et al., 2023). Conversely, pectin-ethylcellulose pellets showed microbially enhanced release *in vitro* that did not translate to pigs, likely because the porcine fecal samples had low pectinase activity (Giakoumis et al., 2026). This discordance shows that a positive batch-fermentation result is a mechanistic signal, not a guarantee of *in vivo* performance.

Results from fermentation models should therefore be reported as one of three evidence levels: confirmation of microbial transformation or degradation; comparative evidence that the formulation changes microbial composition or function under defined conditions; or predictive evidence supported by concordance with more complex models or *in vivo* data. This grading improves reproducibility and prevents a single fecal slurry experiment from being treated as clinical validation.

### 6.2 Dynamic gut models and SHIME-like systems

Dynamic gut models provide a more advanced way to study repeated exposure and regional microbial behavior. Simulator of the Human Intestinal Microbial Ecosystem (SHIME)-like systems are designed to simulate different compartments of the human gastrointestinal microbial ecosystem and have been described as controlled and reproducible *in vitro* platforms (Zhu et al., 2024). For oral formulation development, SHIME-like models can help evaluate how repeated exposure to excipients, surfactants, nanocarriers, or microbiota-responsive materials affect microbial composition and metabolites over time. They are also useful for comparing formulations under more stable microbial community conditions than simple batch models (Subramaniam et al., 2023a).

The major advantage of these systems is that they can support longer experiments, regional simulation, and repeated dosing. This makes them relevant for chronic oral products and high-excipient-load systems. However, SHIME-like systems still do not fully simulate active absorption, peristalsis, epithelial barrier biology, or host immune responses (Zhu et al., 2024). Thus, they should be viewed as intermediate translational screening tools rather than replacements for epithelial models, animal studies, or clinical validation. For microbiota-responsive delivery, dynamic colon models can be especially valuable for testing whether microbial degradation remains reliable over time.

### 6.3 Gut-on-chip and intestinal barrier models

Gut-on-chip systems add host-relevant epithelial and mechanical features that are missing from fermentation-only models. Reviews of gut-on-chip research emphasize their translational value for drug development because they can incorporate intestinal cells, flow, mechanical cues, barrier readouts, and controlled-exposure conditions (Donkers et al., 2023).

Advanced gut-on-chip platforms can incorporate human fecal samples and peristaltic-like movements, creating systems that more closely model host–microbiome interaction (Ballerini et al., 2025). These platforms can measure epithelial barrier markers and cellular responses after exposure to microbiome-containing conditions (Ballerini et al., 2025). Their limitation is complexity. Gut-on-chip models are expensive, technically demanding, less standardized, and offer lower throughput than fermentation or conventional cell culture. Therefore, they are best used after earlier screening has identified a formulation that requires deeper mechanistic or safety evaluation (Valiei et al., 2023).

### 6.4 Organoids and humanized/gnotobiotic animal models

Intestinal organoids are valuable because they can better reflect human epithelial biology and, in some cases, patient-specific or region-specific intestinal features. Current applications of intestinal organoids include disease modeling, drug screening, microbiota studies, and biomolecular delivery (Xiang et al., 2024). Organoids can also support studies of intestinal nutrient transport, drug uptake, and metabolism (Zietek et al., 2020). This makes them useful for testing whether microbiome-exposed formulations influence epithelial absorption or transporter-related endpoints.

The main limitation is that standard three-dimensional organoids often have limited apical access and do not naturally contain a stable complex microbiome unless specifically engineered or co-cultured. A study using laboratory mice engrafted with natural gut microbiota reported a wild-type-like phenotype with greater relevance to human biology (Runge et al., 2025). Organoid monolayers and organoid-on-chip formats can partly address this limitation. Gnotobiotic and humanized microbiota mouse models can then provide causal *in vivo* evidence for defined microbial functions or human microbiome effects (Park and Im, 2020). However, mouse physiology, diet, transit, immune response, and microbiome engraftment differ from humans. These models should therefore be used for mechanistic validation rather than as final proof of human formulation performance (Nguyen et al., 2015).

### 6.5 16S rRNA sequencing, metagenomics, and metabolomics

Microbiome-aware formulation studies should measure both community composition and function. 16S rRNA sequencing is useful for identifying taxonomic shifts, alpha diversity, beta diversity, and major changes in microbial structure. However, 16S alone often cannot determine whether microbial enzyme activity, drug metabolism, or release-trigger capacity has changed (Vanzin et al., 2025).

Functional approaches such as metagenomics and metabolomics are therefore needed when the formulation depends on microbial metabolism or enzyme-mediated release. Reviews of microbiome-related drug liability highlight the importance of functional tools and multi-omics approaches for connecting microbial communities to drug metabolism and translational outcomes (Cai et al., 2023).

Metabolomics is especially important because microbial metabolites can influence barrier function, mucus, inflammation, bile acid pools, and absorption. For formulation studies, relevant outputs may include short-chain fatty acids, bile acids, indoles, phenolic metabolites, drug metabolites, and excipient-derived metabolites (Liu et al., 2022). Sequencing and omics studies should also follow appropriate reporting standards, including minimum information frameworks for marker gene and genomic sequence datasets. Without clear reporting of donor source, exposure conditions, formulation properties, sequencing methods, and analysis pipelines, microbiome data become difficult to compare across studies (Mirzayi et al., 2021).

### 6.6 Minimum microbiome endpoints for oral formulation studies

A practical microbiome-aware testing package should be risk-based rather than universal. Low-risk conventional oral tablets may not require extensive microbiome testing. In contrast, microbiota-responsive systems, surfactant-rich formulations, chronic oral nanocarriers, colon-targeted systems, and formulations intended for dysbiotic or vulnerable patients may require additional endpoints. At minimum, high-relevance studies should measure microbiome composition, microbial functional potential, key metabolites, formulation release or degradation, particle stability, epithelial barrier integrity, and inflammation-related markers. Nanomaterial studies should also document size, dose, exposure duration, and functional groups because these variables can influence gut microbiota outcomes (Utembe et al., 2022).

Overall, the strongest testing strategy is a tiered approach. Early screening can use batch fermentation across multiple donor microbiomes. Promising or concerning formulations can then be tested in SHIME-like or dynamic colon systems. Barrier-relevant formulations should move into Caco-2/HT29-MTX, organoid monolayers, or gut-on-chip systems (Mathur et al., 2026; Takagi et al., 2016). Mechanistic questions about specific microbial functions can be tested in gnotobiotic or humanized microbiota animals. This hierarchy avoids both under-testing and over-testing. It also ensures that microbiome data are linked to formulation-relevant outcomes: release, stability, absorption, barrier safety, and predictable performance. Table 4 summarizes the experimental models for microbiome testing.

**TABLE 4:** Experimental models for microbiome-aware oral formulation testing.

Model	What it measures	Strength	Limitation	Best use case	Minimum endpoints	Key reference (s)
Batch fecal fermentation	Microbial degradation, drug/excipient metabolism, SCFAs, taxa shifts	Fast, low cost, multi-donor screening possible	No epithelial barrier, absorption, immune response, or motility	First-pass microbiome-risk or trigger screen	16S, metagenomics, metabolomics, drug recovery, release	Verhoeckx et al. (2015)
SHIME-like dynamic gut model	Regional and repeated-exposure microbiota composition/function	Controlled, reproducible, multi-compartment exposure	Limited active absorption and host epithelial interaction	Chronic excipient/nanocarrier exposure and metabolite shifts	16S/metagenomics, SCFAs, bile acids, release/degradation	Zhu et al. (2024)
TIM-2/dynamic colon model	Colonic fermentation and microbial metabolism under dynamic conditions	More realistic colon simulation than batch models	Limited epithelial/systemic biology	Testing colon-triggered release and microbial degradation	Drug release, enzyme activity, microbial metabolites	Zhu et al. (2024)
Caco-2/HT29-MTX/Transwell	Permeability, mucus, transepithelial electrical resistance (TEER), epithelial toxicity	Accessible, standardized, useful for barrier screening	Weak microbiome realism and simplified epithelium	Barrier pre-screen for excipients, surfactants, nanocarriers	TEER, tight junctions, permeability, cytotoxicity	Donkers et al. (2023)
Organoid monolayers	Human epithelial transport, uptake, metabolism, patient/region-specific responses	More human-relevant than simple cell lines	Microbiome co-culture is technically difficult	Absorption/metabolism testing after microbiome exposure or formulation contact	Transporters, uptake, metabolites, barrier markers	Xiang et al. (2024), Zietek et al. (2020)
Gut-on-chip	Barrier + flow + mechanical cues + optional microbiome/fecal exposure	High physiological relevance and host–microbe interface potential	Complex, costly, lower throughput, less standardized	Advanced safety/mechanism studies for high-risk formulations	TEER/zonula occludens-1 (ZO-1), cytokines, apoptosis, multi-omics, release/permeability	Donkers et al. (2023), Ballerini et al. (2025)
Gnotobiotic mice	Causal role of defined microbes or enzymes *in vivo*	Controlled microbiome-function validation	Mouse–human differences; simplified communities	Validate enzyme/taxon-specific metabolism or release mechanisms	Pharmacokinetics (PK), tissue exposure, microbiome composition, enzyme activity	Park and Im (2020)
Humanized microbiota mice	Human microbiome effects *in vivo*	Translational bridge beyond *in vitro* models	Human microbiota not perfectly reproduced in mouse physiology	Preclinical validation of patient-like microbiome effects	Pharmacokinetics/pharmacodynamics (PK/PD), microbiome, metabolites, toxicity markers	Park and Im (2020)
Clinical fecal/biopsy samples	Real patient microbiome variability and disease context	Highest translational relevance	Difficult to standardize; ethical/logistical constraints	Final validation of variability and patient stratification	Multi-donor release, metagenomics, metabolites, clinical covariates	Awad et al. (2022)

## 7 Translational challenges and design rules

The evidence reviewed supports the relevance of microbiome-aware oral formulation design, but translation remains limited by fragmented evidence, inconsistent models, and the absence of standardized microbiome-specific formulation testing. The central challenge is not simply whether the microbiome affects oral drug delivery; it is how to decide when this effect is meaningful enough to influence formulation development (Zhang et al., 2021). A risk-based framework is therefore needed to connect drug properties, excipient choice, nanocarrier design, microbial triggers, experimental models, and patient variability.

### 7.1 Lack of standardized microbiome safety testing

Microbiome-related drug effects are increasingly recognized in drug development, but microbiome tools are still not routinely integrated into formulation workflows. A working-group review on gut microbiome integration in small-molecule development emphasized the need to include microbiome-related tools earlier in discovery and development (Jimonet et al., 2024). This is directly relevant to oral drug delivery because orally administered products are most likely to encounter dense microbial communities during transit.

For nanocarriers and nanosized materials, the standardization gap is especially important. A systematic review of nanomaterial effects on gut microbiota concluded that microbiome outcomes depend on variables such as size, dose, exposure duration, and functional groups (Utembe et al., 2022). Therefore, microbiome safety testing should not be separated from pharmaceutical characterization. If nanoparticle size, charge, coating, and exposure duration influence both delivery performance and microbiome effects, these parameters should be interpreted within a single formulation-risk framework.

Regulatory science also supports the relevance of this topic, although it is not yet a dedicated requirement for all oral formulations. The Food and Drug Administration (FDA) recognizes microbiome research as relevant to products that may interact directly or indirectly with microbial communities. FDA nanomaterial guidance also emphasizes careful evaluation of nanomaterial-containing drug products because their attributes may differ from conventional products (U.S. Food and Drug Administration (FDA), 2022). However, current regulatory frameworks do not yet provide a specific microbiome-testing package for oral excipients, SEDDSs, microbiota-responsive systems, or oral nanocarriers. This creates an opportunity for the present review to propose a practical, risk-based testing roadmap rather than claiming that microbiome endpoints are already mandatory (Rodriguez et al., 2025).

### 7.2 Interindividual microbiome variability

Interindividual variability is one of the strongest reasons to include the microbiome in oral formulation thinking. Personalized *ex vivo* studies show that human gut microbial communities differ in their capacity to metabolize drugs (Javdan et al., 2020). This matters because a formulation that performs predictably in standard simulated fluids may behave differently when exposed to patient-specific microbial enzymes, metabolites, or degradation pathways.

This variability becomes more critical when the formulation deliberately depends on microbial activity. Microbiota-responsive systems assume that the required enzyme activity is present at the target site (Kamath et al., 2025). However, bacterial enzymes used for colon-specific delivery have been reported to decrease in active Crohn’s disease. This creates a formulation problem: the very patients who might benefit from colon-targeted delivery may have altered microbial functions that reduce trigger reliability (Crouwel et al., 2021).

The practical implication is that microbiota-responsive formulations should not be validated only in healthy reference microbiota or simplified enzyme media. They should be tested across multiple donor microbiomes and, when relevant, disease-associated microbiota (Metris et al., 2025). Patient factors such as inflammatory bowel disease, antibiotic exposure, diet, age, transit changes, and dysbiosis should be treated as potential modifiers of release. This does not make microbiota-responsive systems unsuitable; it means they should be developed as patient-aware dosage forms (Alexander et al., 2026).

### 7.3 Regulatory and quality-control challenges

A microbiome-aware design framework can be aligned with Quality by Design (QbD) rather than positioned as a separate concept. ICH Q8(R2) defines pharmaceutical development as a systematic approach based on product and process understanding and quality risk management (ICH, 2009). For nanomaterial-containing products, regulatory guidance already emphasizes the need to consider product-specific attributes and quality, safety, and efficacy (U.S. Food and Drug Administration (FDA), 2022). This supports the idea that oral nanocarriers may require additional characterization when their physicochemical properties create microbiome-facing risks. Although microbiome-related attributes are not currently established regulatory critical quality attributes (CQAs) for oral drug products, a QbD-based risk assessment framework may provide a useful approach for evaluating microbiome interactions in formulations where such effects are likely to influence product performance (ICH, 2009).

### 7.4 Microbiome-related CQAs and CMAs

Classical QbD approaches identify critical quality attributes and material attributes that influence product quality and performance (ICH, 2009). For oral lipid nanoparticles, QbD-driven optimization commonly considers attributes such as particle size, polydispersity index, zeta potential, encapsulation efficiency, and release behavior (Hidayat et al., 2025). A microbiome-aware framework does not replace these CQAs; it expands the interpretation to become microbiome-relevant.

For example, particle size and surface chemistry may influence mucus penetration, microbial exposure, and gut microbiota effects (Utembe et al., 2022). Surfactant load may influence dispersion and bioavailability, but it may also affect barrier or microbiome safety in repeated-dose systems. Polysaccharide degradability may be a material attribute when microbial degradation is used for colon-specific release (Azehaf et al., 2023).

The proposed concept is therefore not “microbiome CQAs for every product.” A more defensible approach is to define candidate microbiome-related CQAs, critical material attributes (CMAs), and process considerations only when the formulation has a plausible microbiome-facing mechanism (Duarte et al., 2025; Slijkerman et al., 2025). Examples include microbiota-triggered release consistency, microbiome disruption potential, barrier integrity effect, microbial metabolite shift, surfactant burden, microbiota-degradable material selection, and exposure duration (Kostic et al., 2024).

Across the literature, evidence quality is uneven, and direct comparisons between studies are limited by differences in dose, exposure duration, formulation composition, donor selection, sequencing or analytical workflows, and outcome definitions. Many reported excipient and nanocarrier effects derive from animal, dietary, or simplified *in vitro* exposure rather than clinically matched pharmaceutical use. In disease models, recovery of microbial composition may also follow reduced inflammation rather than direct microbiome modulation by the carrier, whereas a null taxonomic result does not exclude functional or metabolite changes. Systematic reviews of nanomaterial-microbiome studies have highlighted substantial methodological heterogeneity and the need for standardized characterization and testing (Utembe et al., 2022). Moreover, the failure of microbiota-enhanced pectin release observed *in vitro* to translate to pigs demonstrates that a mechanistic signal in one model does not establish predictive formulation performance (Giakoumis et al., 2026). Evidence should therefore be weighted according to exposure relevance, appropriate controls, replication, and concordance across model tiers, rather than the presence of a statistically significant microbiome shift alone.

### 7.5 Proposed microbiome-aware formulation design rules

A practical microbiome-aware design framework should begin with the drug. If the drug is known or suspected to undergo microbial metabolism, formulation design should consider whether it needs protection, altered release location, or microbiome-informed testing (Zimmermann et al., 2019). The second step is excipient classification. Excipients should be assessed as microbiome-compatible, microbiome-active, microbiota-degradable, microbiome-disruptive or barrier-active, microbiome-supportive, or microbiome-rescued, depending on available evidence and formulation context (Subramaniam et al., 2023a).

The third step is nanocarrier interface design. Size, charge, surface chemistry, coating, biodegradability, mucoadhesion, mucus penetration, and surfactant load should be matched to the intended delivery goal rather than optimized generically (Utembe et al., 2022). The fourth step is trigger selection. If microbiota-responsive release is desired, the formulation should be tested under microbiome-relevant conditions, and combined pH-, time-, and enzyme-responsive designs should be considered when trigger variability is expected (Rabeh et al., 2024). The fifth step is model selection. Fermentation, SHIME-like systems, gut-on-chip, organoids, and animal models should be used in a tiered way according to the specific formulation question (Al-Btoosh et al., 2026; Zhu et al., 2024; Zietek et al., 2020).

The following operationalization is presented as the authors’ proposed framework for formulation research and development, not as an established regulatory classification or a mandatory testing guideline.

#### 7.5.1 Step 1: define the drug-microbiome risk

Document known or suspected bacterial transformations, the organism or enzyme when available, the activity and absorbability of the resulting metabolite, the site of transformation relative to absorption, the therapeutic window, dose, and expected treatment duration. The consequence should be classified as intended activation, loss of active exposure, intestinal reactivation or toxicity, or unresolved risk. The resulting decision is whether to protect the drug from microbial contact, deliberately expose it for activation, shift the release location, or proceed with conventional development when no plausible microbiome-facing risk is identified.

#### 7.5.2 Step 2: build an exposure-weighted excipient profile

For each excipient, record the estimated daily amount reaching the colon, duration and frequency of exposure, formulation function, evidence source, dose comparability, and reported microbiome or barrier effects. Evidence from pharmaceutical human exposure, dietary human exposure, animal studies, and *in vitro* systems should not be treated as equivalent. Classification is context-specific: the same excipient may present low concern in a low-dose, short-course immediate-release product and greater concern in a chronic, high-load liquid or self-emulsifying formulation. Selection should preserve required product performance while minimizing plausible microbiome and barrier burden.

#### 7.5.3 Step 3: define the nanocarrier compartment and interface

State whether the carrier is intended to remain in the lumen, adhere to mucus, penetrate mucus, enter epithelial or immune cells, or support systemic absorption. This goal should be translated into target ranges or justified choices for particle size, surface charge, coating, biodegradability, mucoadhesion or mucus penetration, digestion behavior, and surfactant load. A property should not be optimized in isolation: increased positive charge may improve cellular contact while also increasing mucus trapping or microbial-surface interaction. Blank-carrier and free-drug controls are required to separate carrier effects from payload effects.

Surface functionalization studies outside oral delivery can also provide design analogies, provided route-specific limitations are explicit. Hyaluronic acid-decorated quatsomes combined nanoscale size, cationic surface charge, vesicle composition, and ligand functionalization in a renal-targeted curcumin platform that improved biochemical and histological nephroprotection in a cisplatin model (Ahmed S. et al., 2026). Because direct biodistribution was not measured and the study drug was not administered orally, it should be used only as an example of mechanistically informed carrier-interface design, not as evidence for oral or microbiome-specific performance.

#### 7.5.4 Step 4: select and verify the release-trigger architecture

When microbial degradation is used, substrate-specific transformation and drug release should be demonstrated against sterile or microbiota-free controls and tested across multiple donor microbiomes and relevant disease or antibiotic exposure states. If a single trigger is likely to be variable, a pH-, time-, and microbiota-dependent combination or another backup mechanism should be considered (Moutaharrik et al., 2023). A combined system should still distinguish the contribution of each trigger rather than attribute all release to the microbiome.

#### 7.5.5 Step 5: match the testing tier to the development decision

Conventional dissolution and physicochemical testing should establish baseline product behavior; enzyme- or donor-based fermentation should test microbial mechanism and variability; dynamic models should address repeated exposure and regional function; epithelial, organoid, or gut-on-chip systems should evaluate host–interface effects; and animal or clinical studies should be reserved for integrated pharmacokinetic, pharmacodynamic, and safety questions. Advancement criteria should be predefined, including an acceptable release window, donor-to-donor variability, barrier compatibility, and microbiome perturbation threshold. Failure of an *in vitro* microbial trigger to translate *in vivo* should lead to redesign or a revised model strategy rather than *post hoc* reinterpretation (Giakoumis et al., 2026).

#### 7.5.6 Step 6: establish the decision and control strategy

Candidate microbiome-facing critical quality or material attributes should be defined only when a plausible mechanism links them to product performance or safety. The complete formulation should be compared with the free-drug, blank-carrier, and excipient-matched controls, and residual uncertainty should be documented. If performance depends on unstable donor enzyme activity or the formulation causes reproducible adverse changes at relevant exposure, the material, excipient load, trigger architecture, or release site should be redesigned. If no meaningful microbiome-facing signal is detected, that conclusion should also be documented so unnecessary testing is not continued. The assessment should be revisited during scale-up because processing can change particle properties, digestion, and release behavior.

Overall, microbiome-aware formulation design should be risk-based and goal-specific. It should not add unnecessary testing to every oral product but should prevent microbiome-relevant risks from being ignored in formulations where microbial metabolism, excipient effects, nanocarrier properties, or microbiota-triggered release could influence performance (Metris et al., 2025). This design-rule framework is the main translational output of the review and provides a bridge to future precision oral pharmaceutics. Figure 4 illustrates the decision framework linking formulation properties to *in vivo* performance.

**FIGURE 4:**
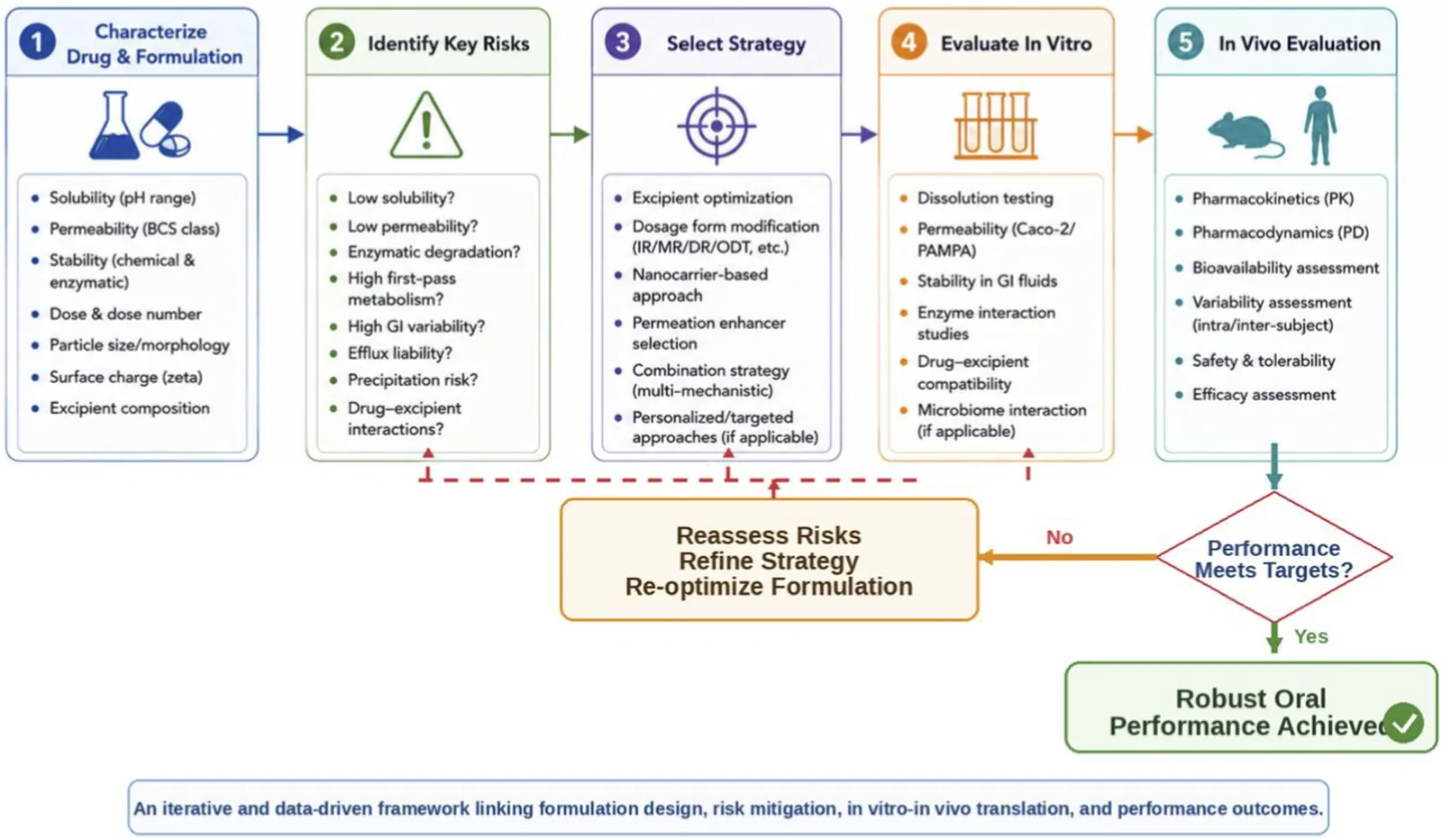
Decision framework linking formulation properties to *in vivo* performance. The workflow begins with drug and formulation characterization, proceeds through risk identification, strategy selection, *in vitro* evaluation, and *in vivo* assessment, and uses iterative feedback to refine the formulation until robust oral performance is achieved.

### 7.6 Clinical translation and feasibility of implementation

Clinical translation should be selective and tied to a specific development decision. Microbiome-aware testing is most feasible when introduced as an early risk screen for products with a plausible microbiome-facing mechanism, including known microbial drug metabolism, high or repeated luminal excipient exposure, surfactant-rich systems, local intestinal nanocarriers, and microbiota-dependent release. A positive signal should lead to a defined formulation action, such as reducing an excipient load, changing the carrier or coating, relocating release, or adding a second trigger, rather than generating descriptive microbiome data without a development consequence. Working-group recommendations for small-molecule development similarly emphasize fit-for-purpose microbial stability, fermentation, model, clinical, and regulatory tools rather than universal testing (Jimonet et al., 2024).

Regulatory implementation remains challenging because no generally established microbiome-specific testing package for conventional oral formulations currently exists. A practical route is to incorporate justified microbiome-facing risks into existing pharmaceutical development and quality–risk frameworks, document the relationship between the tested exposure and the intended clinical dose, and predefine acceptance or redesign criteria. Translation of advanced colonic systems further requires scalable manufacture, reproducible release in the target population, and evidence that *in vitro* or preclinical trigger performance is retained clinically (Awad et al., 2022).

Patient stratification is currently most plausible for a limited group of products whose performance depends on a measurable microbial function. Candidate stratifiers could include the abundance or activity of a drug-metabolizing enzyme, recent antibiotic exposure, disease-associated dysbiosis, or a validated metabolite signature. However, routine microbiome-guided formulation selection is not yet clinically established. Individualized *ex vivo* studies demonstrate substantial patient-to-patient differences in microbial drug metabolism, but prospective evidence is still needed to define actionable thresholds and show that stratification improves efficacy or safety.

## 8 Future perspectives

Microbiome-aware oral drug delivery is still an emerging formulation concept. Its future value will depend on whether the field can move from descriptive microbiome associations toward predictive, testable, and formulation-relevant decision making. The most important next step is not simply to generate more microbiome data but to connect microbiome data to drug release, stability, absorption, safety, and patient variability (Zai, 2026).

### 8.1 Precision pharmaceutics and microbiome stratification

A major future direction is patient microbiome stratification. Reviews on microbiota-targeted precision medicine highlight that the gut microbiota varies substantially between individuals and may influence drug efficacy and toxicity (Feng et al., 2020). For oral formulation design, this opens the possibility of stratifying patients according to microbial enzyme activity, microbial drug-metabolizing capacity, dysbiosis status, or recent antibiotic exposure. This would be especially relevant for microbiota-responsive systems, drugs with known microbial metabolism, and colon-targeted therapies for inflammatory bowel disease (Bashiardes and Christodoulou, 2024).

Patient disease context can alter the gastrointestinal environment in ways that complicate formulation prediction. An integrative review of Huntington disease describes gastrointestinal dysmotility, barrier dysfunction, altered gut microbial composition, and changes in microbiota-derived metabolites as components of the disease’s multisystem pathology (Senousy et al., 2026). This is not evidence of a formulation–microbiome interaction; rather, it illustrates why disease state may need to be considered when interpreting oral formulation performance and interpatient variability.

This idea should be framed as a future direction rather than a current clinical standard. Routine microbiome-based selection of oral formulations is not yet established. However, the concept is scientifically plausible because patient-specific microbiomes can differ in drug-metabolizing capacity (Javdan et al., 2020). Future development may therefore include microbiome screening as part of formulation selection for high-risk or high-opportunity oral products.

### 8.2 Microbiome-informed excipient selection

Another important future direction is microbiome-informed excipient selection. Current formulation development typically prioritizes solubility, stability, manufacturability, taste, coating performance, and regulatory acceptability. These criteria remain essential, but excipient–microbiome compatibility may become an additional screen for selected oral products. Excipients can directly interact with gut microbes (Subramaniam et al., 2023a), and some food and drug additives can influence intestinal drug transport (Zou et al., 2020). These findings support more data-driven excipient selection when microbiome relevance is plausible.

This does not mean that every excipient requires full microbiome profiling. A risk-based approach is more practical. Microbiome-informed excipient screening is most relevant for chronic oral medicines, pediatric or geriatric formulations, inflammatory bowel disease (IBD) products, high-load surfactant systems, microbiota-responsive dosage forms, and formulations intended for dysbiotic or vulnerable patients (Subramaniam et al., 2023a). Future studies should define exposure thresholds, dose relevance, and standardized endpoints so that excipient–microbiome effects can be interpreted in pharmaceutical rather than only dietary contexts.

### 8.3 Next-generation microbiota-responsive nanocarriers

Next-generation microbiota-responsive nanocarriers should combine microbial triggers with rational nanocarrier engineering. Natural polysaccharides remain important because they can exploit microbial degradation for colon-targeted release (Azehaf et al., 2023). However, future systems should go beyond simple degradation concepts by integrating pH protection, time control, microbial enzyme responsiveness, mucus interaction, particle size, surface charge, coating design, and microbiome variability.

Multi-trigger systems are especially promising because they can reduce dependence on a single variable. For example, combining pH responsiveness with microbial enzyme sensitivity may improve robustness when either gastrointestinal pH or microbial activity varies (Rabeh et al., 2024). Emerging systems should also be tested using multi-donor microbiota and disease-relevant models. A microbiota-responsive nanocarrier should both release the drug in idealized enzyme media and demonstrate reliable release across microbiome conditions that resemble the intended patient population.

### 8.4 AI-assisted formulation design and microbiome profiling

AI-assisted formulation design could integrate drug properties, excipient identity and exposure, nanocarrier attributes, microbial genes or enzyme activity, metabolite profiles, and release or barrier outcomes to prioritize formulation strategies and testing needs. A recently reported data-driven and knowledge-guided platform illustrates the potential of artificial intelligence to support formulation-strategy selection across oral and injectable products (Wang et al., 2025).

For microbiome-aware products, predictive models could combine taxonomic, functional, and metabolomic profiles with formulation variables to identify responder groups or variable microbial triggers. However, current microbiome machine-learning research remains constrained by heterogeneous datasets, high dimensionality, and limited external and prospective validation (Malakar et al., 2024). AI should therefore be presented as a future tool for risk ranking, hypothesis generation, and experiment selection rather than a validated clinical decision system. Standardized datasets and prospective validation are prerequisites for personalized implementation.

### 8.5 What must happen before clinical translation

Before clinical translation, the field needs standardized microbiome-aware formulation testing, better integration of functional omics, and stronger links between microbiome endpoints and formulation performance. Nanomaterial studies already show that microbiome outcomes depend on size, dose, duration, and functional groups (Utembe et al., 2022). These variables should be reported consistently in oral nanocarrier studies.

For colon-targeted delivery, clinical translation reviews emphasize that advanced colonic drug delivery technologies still face challenges related to targeting reliability, patient variability, and validation (Awad et al., 2022).

Poorly soluble natural products illustrate another gap between formulation innovation and clinical translation. A recent review of baicalin describes liposomes, solid lipid nanoparticles, polymeric nanoparticles, and surface-modified platforms as improving preclinical pharmacokinetic performance, while emphasizing that clinical validation remains limited (Ahmed M. K. et al., 2026). This example reinforces that improved formulation performance alone does not establish clinical translation or microbiome compatibility.

The future of microbiome-aware oral drug delivery should therefore be built around three principles. First, microbiome testing should be used selectively where it can inform formulation decisions. Second, microbiome endpoints should be functional (including enzyme activity, metabolites, and release behavior) beyond compositional profiling. Third, design rules should remain evidence-calibrated: strong claims require human or clinically relevant data, while preclinical findings should guide hypotheses and testing priorities (Kamath et al., 2023). If these principles are followed, microbiome-aware formulation design could improve the predictability, safety, and patient relevance of oral dosage forms.

## 9 Current limitations and knowledge gaps

The current evidence base is uneven. Clinically established examples of microbial drug metabolism coexist with formulation-specific evidence that is largely derived from *in vitro*, animal, or dietary-exposure studies. Exposures, carrier compositions, treatment durations, and microbiome endpoints differ substantially across studies, limiting quantitative comparison and preventing direct extrapolation to routine pharmaceutical dosing.

Systematic evaluation of nanomaterial-microbiome studies has likewise identified heterogeneity in material characterization, dosing, test systems, and reporting (Utembe et al., 2022).

Patient variability remains a major limitation. Microbial composition and functional capacity vary with disease, diet, age, geography, intestinal transit, and antibiotic exposure; consequently, no single donor or pooled inoculum can establish population-wide trigger reliability. Personalized *ex vivo* mapping has demonstrated marked interindividual differences in microbial drug metabolism (Javdan et al., 2020), but comparable prospective evidence linking microbiome profiles to formulation release, efficacy, or toxicity remains limited.

Experimental models reproduce different portions of the formulation–microbiome–host interface. Fermentation models can demonstrate microbial transformation but lack absorption and host feedback; epithelial and organoid models provide barrier information but usually simplify microbial ecology; animal models permit integrated assessment but differ from humans in physiology, diet, transit, and microbiome function. These limitations make prediction across model tiers uncertain, particularly for microbiota-responsive systems. Clinical translation studies of advanced colonic delivery technologies emphasize the continuing need to demonstrate scalable manufacture, reliable targeting, and performance in relevant patient populations (Awad et al., 2022)

## 10 Conclusion

The gut microbiome is a formulation-relevant factor in oral drug metabolism and interpatient variability, as demonstrated by direct microbial drug-metabolism studies (Zimmermann et al., 2019) and personalized *ex vivo* mapping (Javdan et al., 2020). Evidence reviewed here also indicates potential formulation-specific effects on release and barrier exposure, although much of this evidence remains preclinical and context dependent. These findings support microbiome assessment when a plausible mechanism could influence product performance, rather than routine testing of every oral formulation.

This review proposes an authors’ evidence-informed framework that links excipient exposure (Subramaniam et al., 2023a), additive effects (Zou et al., 2020), nanocarrier properties (Utembe et al., 2022), and microbiota-responsive materials (Azehaf et al., 2023) to risk-based formulation decisions. Clinical translation will require pharmaceutical-dose human evidence, reproducible multi-donor testing, standardized functional endpoints, and validation in relevant patient populations.
